# Advanced Design of Soft Robots with Artificial Intelligence

**DOI:** 10.1007/s40820-024-01423-3

**Published:** 2024-06-13

**Authors:** Ying Cao, Bingang Xu, Bin Li, Hong Fu

**Affiliations:** 1 Nanotechnology Center, School of Fashion and Textiles, The Hong Kong Polytechnic University, Hong Kong, 999077 People’s Republic of China; 2 Department of Mathematics and Information Technology, The Education University of Hong Kong, Hong Kong, 999077 People’s Republic of China; 3 Bioinspired Engineering and Biomechanics Center, Xi’an Jiaotong University, Xi’an, 710049 People’s Republic of China

**Keywords:** Soft robotic systems, Artificial intelligence, Design tactics, Review and perspective

## Abstract

- A comprehensive review focused on the whole systems of the soft robotics with artificial intelligence, which can feel, think, react and interact with humans, is presented.
- The design strategies concerning about various aspects of the soft robotics, like component materials, device structures, prepared technologies, integrated method, and potential applications, are summarized.
- A broad outlook on the future considerations for the soft robots is proposed.

## Abstract (highlights)

In recent years, breakthrough has been made in the field of artificial intelligence (AI), which has also revolutionized the industry of robotics. Soft robots featured with high-level safety, less weight, lower power consumption have always been one of the research hotspots. Recently, multifunctional sensors for perception of soft robotics have been rapidly developed, while more algorithms and models of machine learning with high accuracy have been optimized and proposed. Designs of soft robots with AI have also been advanced ranging from multimodal sensing, human–machine interaction to effective actuation in robotic systems. Nonetheless, comprehensive reviews concerning the new developments and strategies for the ingenious design of the soft robotic systems equipped with AI are rare. Here, the new development is systematically reviewed in the field of soft robots with AI. First, background and mechanisms of soft robotic systems are briefed, after which development focused on how to endow the soft robots with AI, including the aspects of feeling, thought and reaction, is illustrated. Next, applications of soft robots with AI are systematically summarized and discussed together with advanced strategies proposed for performance enhancement. Design thoughts for future intelligent soft robotics are pointed out. Finally, some perspectives are put forward.

## Graphical abstract

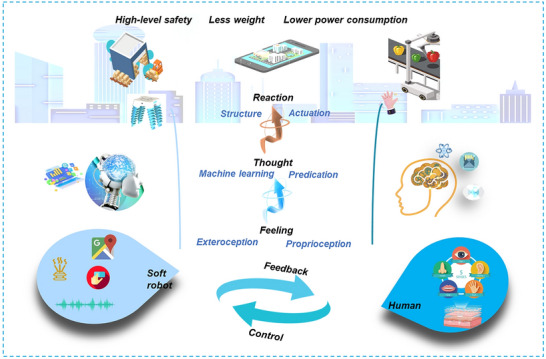

## Introduction

Recent years have witnessed the fascinating changes and the tremendous convenience brought about by the artificial intelligence (AI), including the emergence of ChatGPT which is an AI-powered language model and can generate human-like text flexibly according to the context of situation [1–11]. AI which can make a computer or robot to think intelligently like human is believed to be one of the most exciting advancements in the world. Meanwhile, the internet becomes more easily accessible owing to the rollout of the 5G, and rapid progresses have also been made in the Internet of Things (IoTs) all over the world [12]. The rapid development of AI and IoTs has also revolutionized many relevant fields, among which the robotics has attracted much attention. The AI-enhanced data analytic capability powerfully enhances the functionalities of the sensors for the robots, while the advancements of artificial intelligence of things (AIoT) accelerate development of multifunctional sensors in the robotic systems to extract abundant sensory information. Besides sensors, the AIoT technology has been introduced into the advanced design and fabrication of human–machine interactions and intelligent actuators as well.

Inspired by the physically adaptive and reconfigurable of animal limbs and human muscles, the soft robotic systems based on soft materials are developed [13]. On one hand, the softness of these actuators makes it possible for them to collect and assemble some fragile objects in unmanned factories. One the other hand, they are also managed to be integrated into wearable devices to offer assistance to people with user comfort. For the latter aspect, the soft robots can be qualified for autonomously extending or retracting garments [14], devices for haptics [14], neuroprosthetic hand [15], and so on.

For instance, with the increased degrees of freedom, it is possible for the soft robots to perform unprecedented adaptation to dynamic environments [16, 17]. Moreover, the soft robots are very crucial for some extreme conditions or contaminated environments for the reason that they can get access to some hazardous compounds that are dangerous to humans, realizing their great values in the fields of agricultural development, environmental protection and security surveillance [18]. What is more, all the world has experienced the threat of the COVID-19 pandemic, and therefore, there is a high demand for the autonomous detection toward hazardous substance and virus, which can also be accomplished by the soft robots with both physical and chemical sensing abilities.

**Fig. 1:**
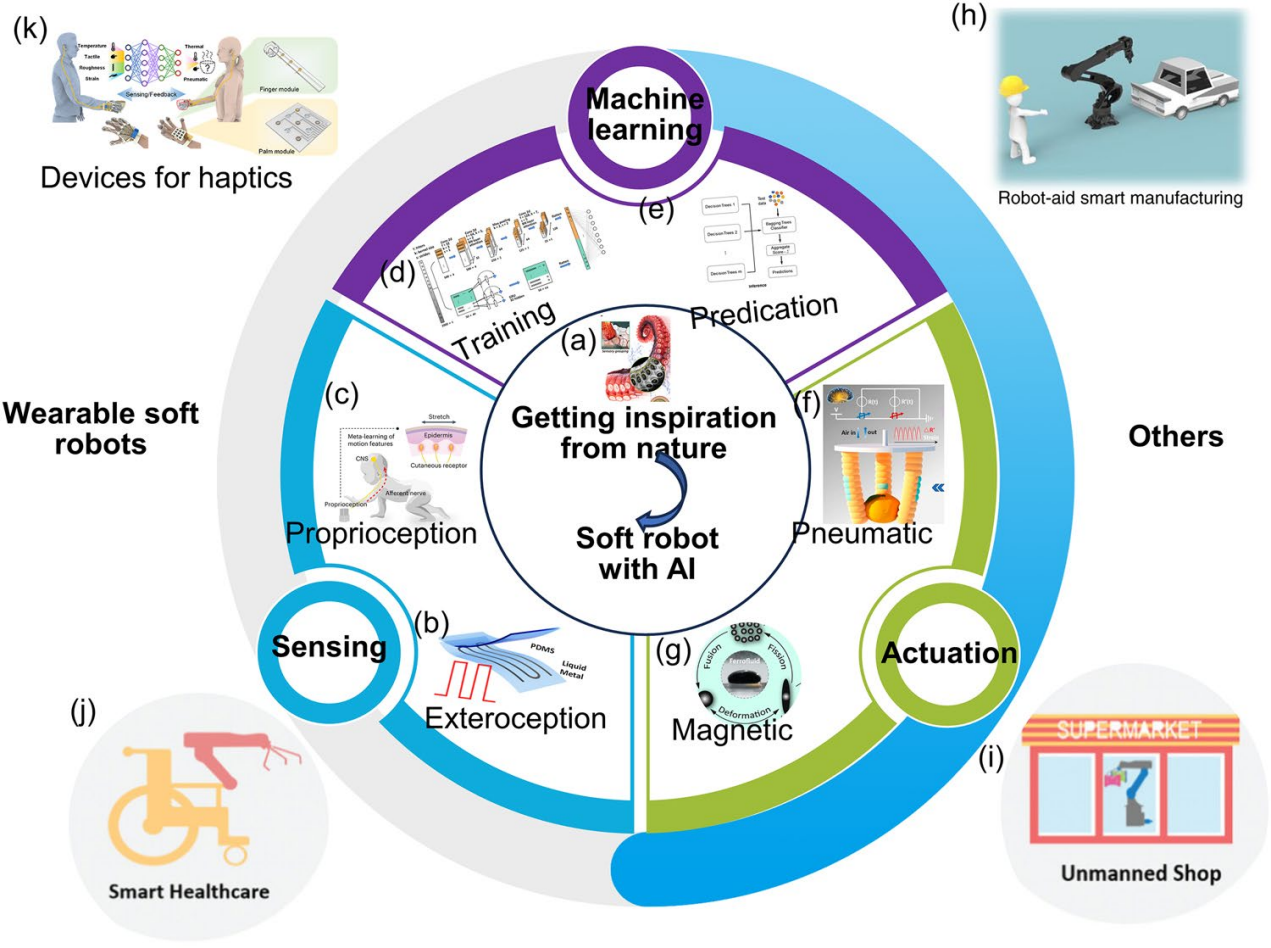
Overview of the soft robots equipped with artificial intelligence (AI)

It is noticeable that when introducing AI into soft robotic systems, the soft robots can be good at self-learning, problem-solving and decision-making which are important features for self-regulating behaviors, and are qualified to be applied in the dynamic environment of the real world (Fig. 1). Despite of the fact that the introducing of AI into the traditional rigid robots can also bring about a lot of advantages, the combination of AI and soft robots can realize many other effects which cannot accomplished by rigid robots with AI. Compared with their counterparts of the traditional robots fabricated from the rigid materials, the soft robots have the advantages of compliance, dexterity, softness, deformability, high-level safety, less weight, lower power consumption, less manufacturing costs and safer contact to live tissues, which have been regarded as the bridge between machines and biological organisms [19–25]. In contrast to the rigid robots which are more commonly used in the industries, it is much easier for the soft robots to offer elaborated services and accomplish delicate tasks owing to the desirable features of lightweight, flexibility, excellent conformability and so on [12, 26–31]. The soft robots demonstrate great potentials in many fields, ranging from IoT to extreme environment operations [32–35]. Furthermore, when machine learning (ML) is introduced into soft robots, the systems are equipped with artificial intelligence to realize the function of biological organisms to the largest extent [23]. For instance, the intelligent soft robots are expected to conduct some surgery in narrow spaces with more security which cannot be accomplished by the rigid robots.

It is noticeable that some small differences in signals neglected by humans can also be detected by ML. A diversity of functions like real-time object classification, gesture estimation, and touch modality recognition have already been achieved by the soft robots with the assist of ML.

Recent years have witnessed a rapid development of the intelligent soft robots. Understanding in biology and bionics has paved the way for the research of smart soft robots, and it has aroused interdisciplinary interest, including chemistry, materials engineering, wearable technology, intelligent manufacturing, and advanced fabrication [36–49]. Some soft robotics with both the proprioceptive sense for the reconstruction of their shapes and exteroception for the identification of various terrains have been successfully developed [23]. Abundant perceptions, such as tactile recognition, temperature sensitivity, visual navigation and even detection for some hazard chemicals or explosives, which are of great importance for realizing environment awareness and threat recognition, have been endowed to the smart soft robots [50]. The development of multisensory perception, close-loop human–machine interaction, ML capability and adaptable execution in one compact system has been realized in some cases, which can be exploited in virtual shop and unmanned factory. It is also possible for the intelligent soft robots which have been developed recently to work in complex underwater environments where the visibility is poor and the spaces are narrow with the ability to recognize multiple contact positions for grasping vulnerable objects [51].

**Fig. 2:**
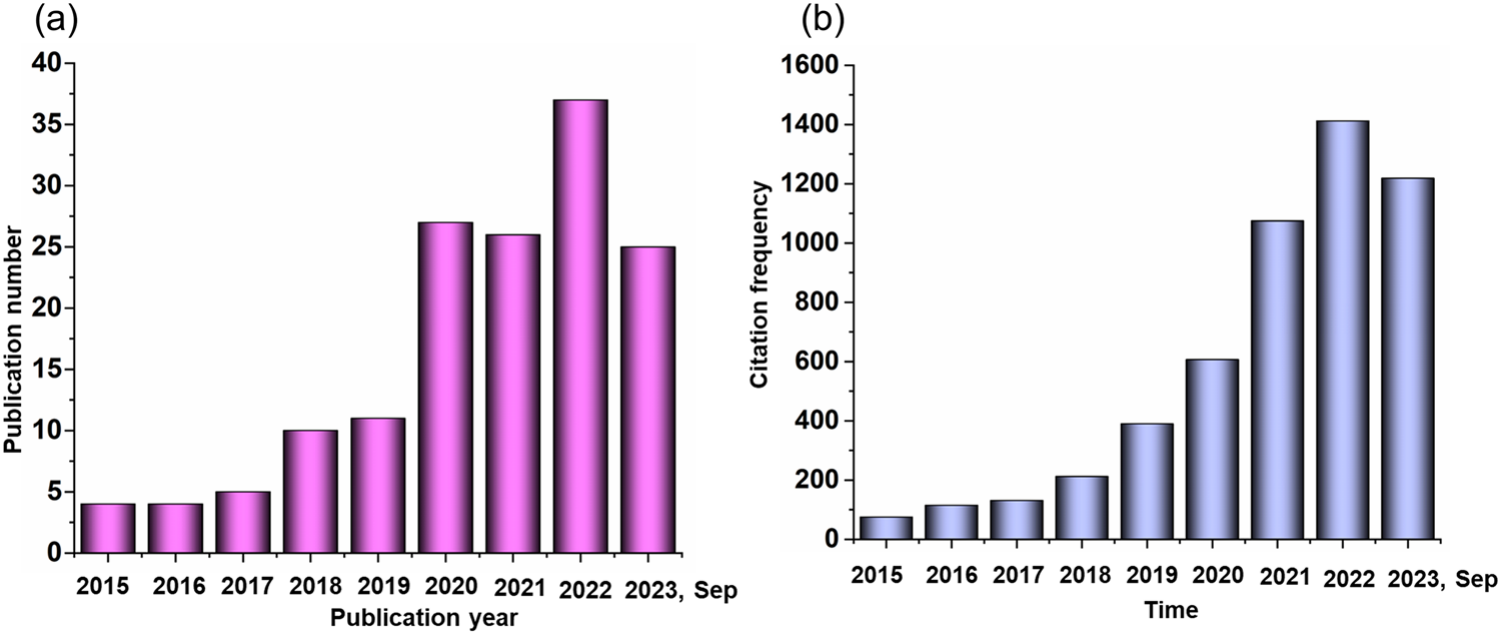
**a** Publication focused on the soft robotics with AI. **b** The citation frequency of the papers concerning about the intelligent soft robots in each year

**Fig. 3:**
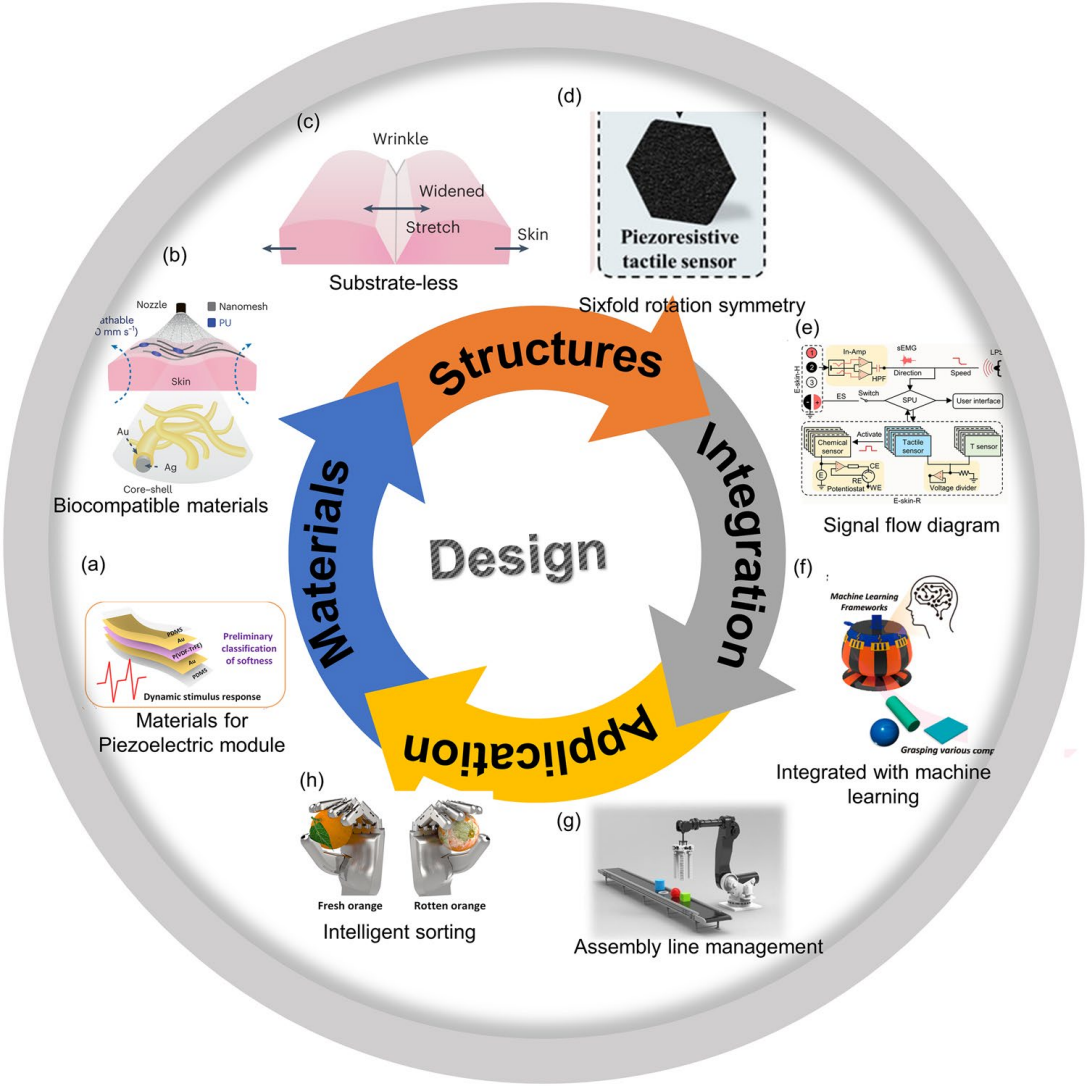
Design tactics related to the soft robots with AI

As a result, many original works of high quality have been published with the citations growing sharply over time, which is clearly illustrated in Fig. 2. However, the overall development of the whole soft robotic systems with AI has not been summarized in its entirety. Herein, this review focused on the advanced design of the intelligent soft robotic systems related to the raw materials, functional devices, fabricated technologies, integration strategies and application scenarios (Fig. 3). Specifically, the basic background of the soft robots with AI was introduced, including the entire working systems of the intelligent soft robots, the mechanisms of machine learning and the actuation method of soft robots. Subsequently, the means by which the soft robots could be equipped with AI was outlined, which was mainly discussed from the aspects of feeling, thought and reaction. The typical application scenarios of the intelligent soft robots were demonstrated, and the strategies to enhance the overall performance of the smart soft robots were discussed. Design considerations for the future soft robots with AI were also proposed. Last but not least, we come up with some ideas and suggestions with respect to the outlook of the soft robotics equipped with AI.

## Mechanisms for Execution of Soft Robots

### Cooperative Work Systems of Soft Robots

**Fig. 4:**
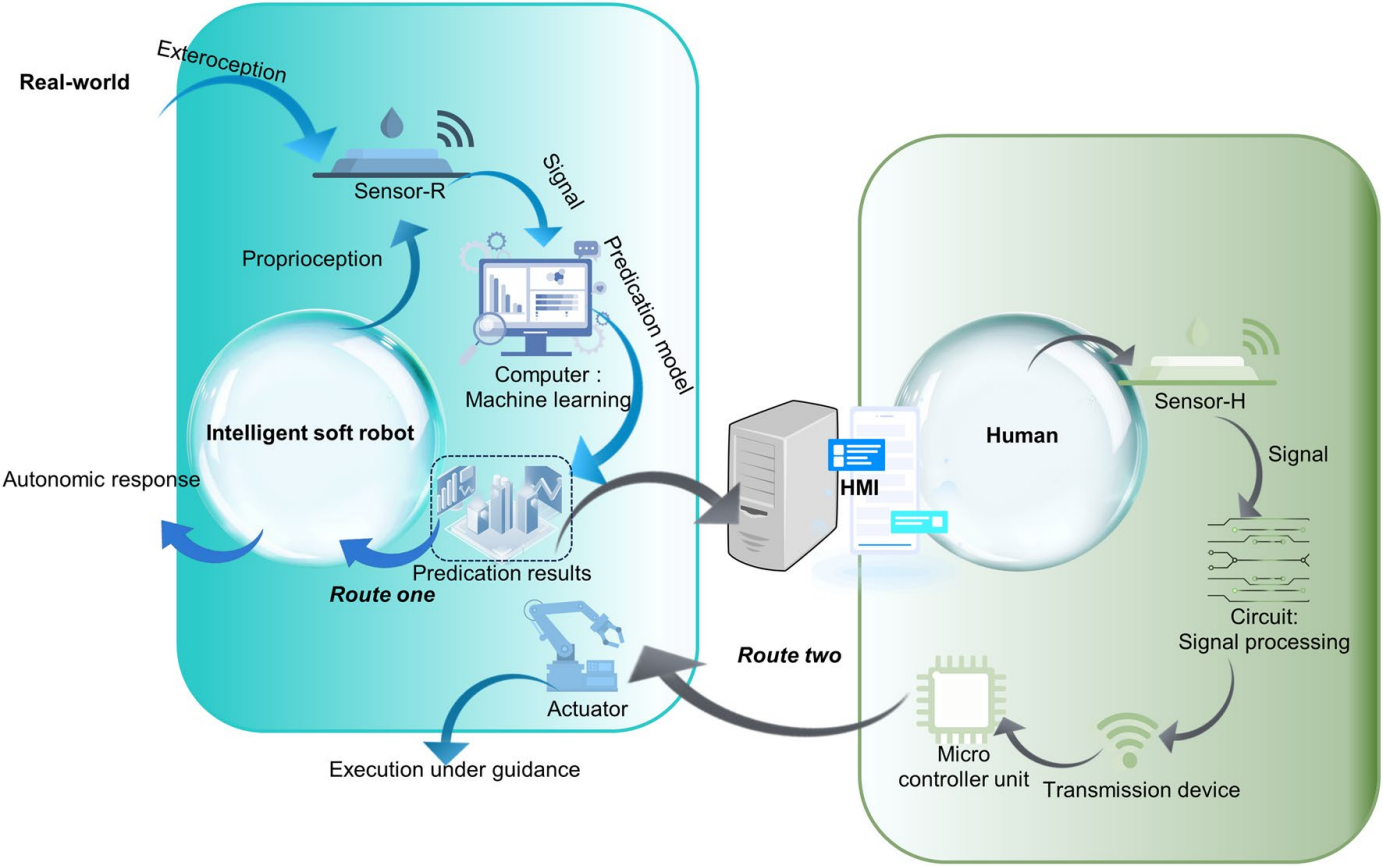
Schematic illustration for the working systems of the intelligent soft robots

The sophisticatedly programmable soft robotic systems with AI are composed of various parts to realize the sensitive perception, optimized learning, closed-loop interactions and excellent adaptability (Fig. 4). In an intelligent soft robotic system, multifunctional sensors on the soft robot (sensor-R) can receive external stimuli (exteroception) from the real world, while it is also managed to monitor the shape of itself (proprioception) [50, 52–57]. The signals or data collected are then processed via ML to obtain a predication result about the object detected by the sensors. For some simple intelligent systems, autonomic response occurs, and the soft robots can implement some tasks like grasping. For the other soft robotic systems, the predication result can be displayed on the human–machine interface (HMI). After that, the operators can give instructions to the soft robots. The human motions can be detected by the sensors integrated with the human (sensor-H), after which processing and transmission of the signals are carried out. The signals are then processed by the microcontroller unit and interpreted by the controller module. The soft actuators of the robots are then worked according to the human intention to accomplish tasks, while the operation status can be also be demonstrated clearly in real-time.

### Mechanisms of Sensors

**Fig. 5:**
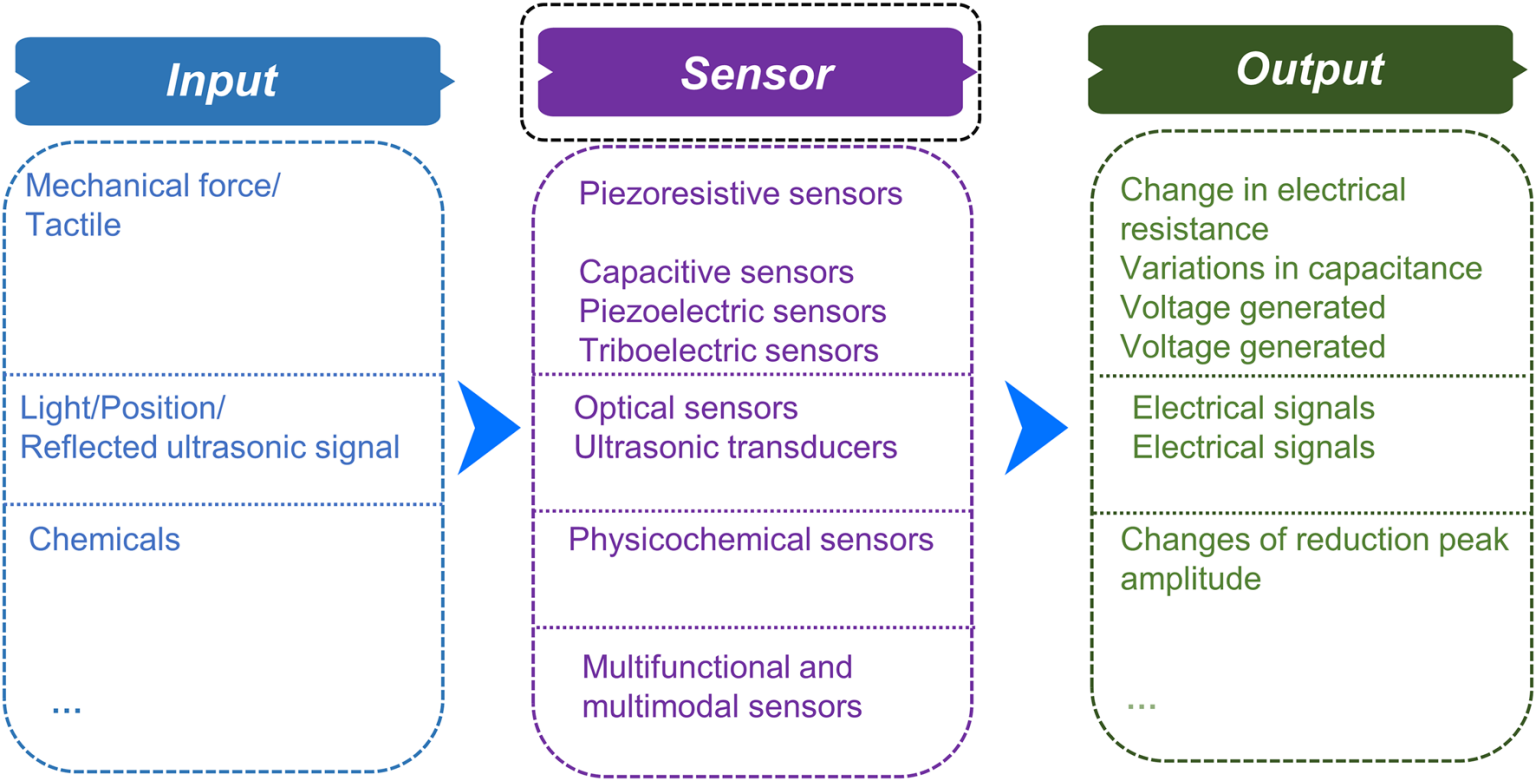
Schematic illustration for the working mechanism of sensors

A diversity of sensors is developed to convert the input information from the environment in which the soft robots are operated to the output signal that can be recoded. The input signals include various information reflected their work environment, including but not limited to the tactile information, the visual pattern and the olfactory signal (Fig. 5). The sensors are expected to be sensitive enough in order to fully convey the information for the autonomous action of the soft robotics or for the awareness of the humans to have a better knowledge of the operated states of the soft robotic systems.

The tactile perception is important for the realization of environment awareness. The mechanical stimuli upon touching the objects can be mainly converted into electrical signals. According to the mechanism, the tactile sensors can be mainly classified into piezoresistive, capacitive, piezoelectric, triboelectric, etc. As for piezoresistive sensors, the electrical resistance changes via the piezoresistive effect [58]. For a capacitive sensor, variations in capacitance happen for detecting mechanical signals [59]. In regard to piezoelectric and triboelectric mechanical sensors, mechanical stimuli can be reflected by the generated voltage. To be specific, electrical signal can be generated when strain or pressure is exerted on the piezoelectric materials, while electric can be produced by means of contact, separation or friction between two materials for triboelectric mechanical sensors [60, 61].

Besides the tactile detection, other stimuli are also expected for the intelligent robots to be precepted so as to collect the information comprehensively and respond accordingly. In order to endow the systems with visual capabilities, cameras or optical sensors are always integrated onto the soft robots. In addition to cameras or optical sensors, some sensors based on the ultrasonic transducers are also applied, since they can emit and receive the reflected ultrasonic waves to detect the position of the objects [62]. Toxic chemicals can be detected by the chemical sensors, in which the reaction between the components in the sensors and the hazard chemicals can be reflected by some specific technology, like negative differential pulse voltammetry (nDPV), alongside with the changes of the peak amplitude [18]. It is noticeable that multi-functional sensors are also in high demand to provide the complex information in the environment.

### Mechanisms of Machine Learning

**Fig. 6:**
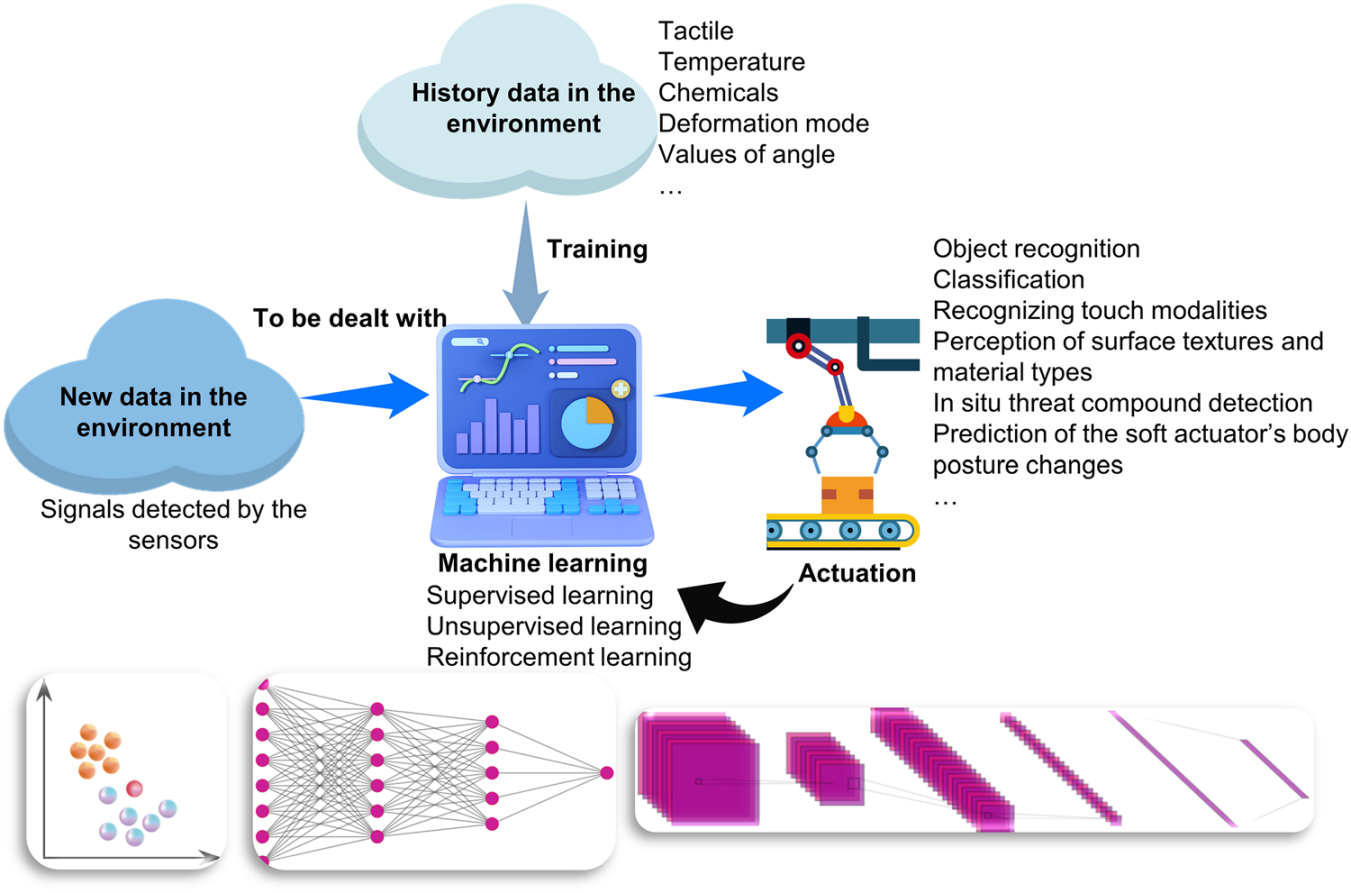
Schematic illustration for the basic model of machine learning in the intelligent soft robotic system

As is known, humans are capable of summarizing the experience into rules, which can then be taken advantage of to make predications when they are encountered with new problems. Accordingly, the soft robots with AI are also managed to learn from the history data and deal with new situations. In a typical ML process, firstly, the vast amount of history data in the working environment detected by the sensors can be learned, during which process a variety of algorithms can be selected for extracting useful information from large quantities of data. Inspired by biological processes, more suitable algorithms and architectures for analogous tasks in soft robots can be developed [63]. It is during the learning process that the information is processed to knowledge which is put into the repository [64]. Since various information is provided by the external environment to the learning systems, the quality of information can exert an important effect on the complexity of the learning realization. Many general principles stored in the repository can then be used to guide the implementation of a certain task when new data in the environment are provided for the machine to be dealt with. It is worthwhile mentioning that the feedback information can also be used to learn and to guide the further study (Fig. 6).

ML can be classified into supervised learning, unsupervised learning and reinforcement learning [65]. For supervised learning, there are both input and output variables with an algorithm helping to understand the mapping function from input to output. In contrast to supervised learning, only input is included in the unsupervised learning. It is noticeable that the data in supervised learning are labeled, while that in unsupervised learning is un-labeled. In the aspect of accuracy, which is of great importance in ML, more reliable and accurate results can be obtained by supervised learning, while average results are provided by unsupervised learning. As to reinforcement learning, it refers to a system that takes actions based on its interaction with the environment and tries to receive the highest reward.

In the soft robotic system, the data collected by the sensors are applied as input, such as tactile, temperature, chemicals and so on. Various goals can be reached by the soft robots by making use of ML, including but not limited to object recognition, classification, detection of touch modalities and perception of surface textures and material types. ML algorithms are able to learn from experiences without being programmed. For instance, k Nearest Neighbor (kNN) algorithm is a classification algorithm, as data points are grouped into several classes, and this algorithm tries to classify the sample data point supplied to it [66]. This algorithm is features with simpleness and easy implementation. The deep neural network (DNN), which is also known as a feedforward network, is an artificial neural network (ANN) with multiple fully connected layers inside it [67–71]. The features are extracted from the data, and the most optimal way by which the input can be converted into the output is acquired. A significant amount of data is worked on by the DNN. Convolutional neural network (CNN) is another deep network that is inspired by visual perception and managed to extract features from data with convolution structures [72–76]. CNN has a series of advantages. For example, each neuron in CNN is connected to only a small number of neurons of the previous layer, as a result of which parameters are reduced and convergence is speeded up [77].

### Mechanisms of the Actuation

**Fig. 7:**
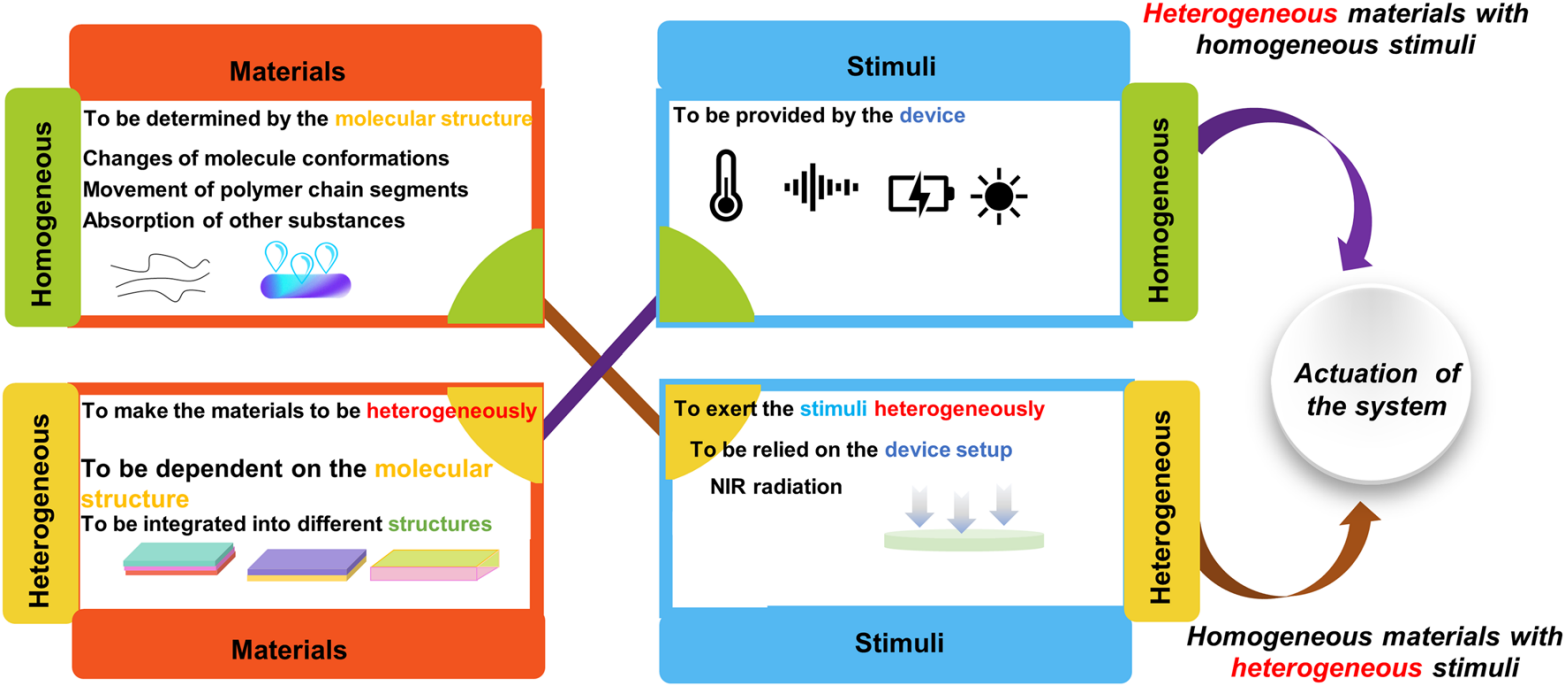
Schematic illustration for the mechanism of actuation in the intelligent soft robotic system

The actuations of the soft robots are mainly focused on how to make the deformation or locomotion under control to accomplish specific tasks, and therefore, the mechanism of soft actuators is explained based on the mechanisms for deformations of materials and control of the deformations (Fig. 7).

Mechanisms for the deformations of the materials can be illustrated from two aspects, including materials and stimuli, which means that deformation can be induced when certain kinds of stimuli are imposed onto the materials. The deep mechanism underlying the deformation can be derived from the microlevel, such as changes of molecule conformations, movement of polymer chain segments and absorption of other substances.

Deformations occur when molecule conformations change. The elastomers that are commonly applied in the soft robots are amorphous three-dimensional polymer networks with the operating temperature above their glass transition temperature. The deformations of elastomers which are mostly entropic can happen upon various stimuli, during which period the conformation of molecules changes, while the distance between atoms/molecules remains almost the same. As for elastomers, the dependence of engineering stress on strain is not strictly linear. The elastomers can undergo a fully reversible deformation when exposed to a force, during which process polymer chains change from the equilibrium conformation of the random coil to a nonequilibrium stretched one [78–84]. In addition, the elastic modulus which can reflect the deformability of the elastomers is in close relationship with cross-linking density and temperature [85]. Accordingly, several kinds of stimuli which can provide force for the systems are effective to induce the deformations of elastomers. The force can be provided directly, while it can also be provided by other methods. For instance, the easiest way to provide force is to make the elastomers be stretched either manually or by motors. Besides, inflation is also a common approach to supply force for the actuation of the soft robots. In addition to these direct ways, force can also be generated by electric, magnetic fields and so on [86]. For the force provided indirectly, the actuations realize either based on the other properties of elastomers themselves like dielectric elastomer or relied on some key features introduced by the other materials like magnetic particles existing in the matrix of the elastomers. For example, the dielectric elastomer is a type of electro-responsive polymer [87–90]. When a dielectric elastomer is sandwiched between two electrodes, it can work as an electric capacitor in an electrostatic setup. After electric potentials are exerted to the electrodes, an electrostatic attraction force between electrodes can then be generated, making the dielectric elastomer being compressed. Besides, when the magnetic particles are introduced into the elastomers, force can be generated by the exposure to magnetic fields [91–95].

Deformations also occur when the movements of polymer chain segments exist. The polymer chain segments can move upon being heated above the glass transition/melting point, which will then lead to the shape changes. Many shape memory polymers (SMPs) are based on this mechanism. In a frozen state, SMPs behave as usual polymers that are below a glass transition temperature/crystallization temperature, while they are soft and perform as elastomers above their glass transition/meting point. As a result, the relations developed for elastomers can also be suitable to shape-memory polymers [80, 84].

Additionally, the absorption of other substances is also a reason that can induce the deformations of the materials. Gels with polymer networks that contain an extensive amount of solvent can have profound swelling and shrinking [96–98]. For instance, the actuation of hydrogel can be realized with the solvent mass transport inside and from the polymer networks [99]. The amount of absorbed solvent (swelling degree) depends on the balance between the elastic deformation of chains and the mixing energy, which consists of both enthalpic and entropic components and can be described by Flory theory. According to Flory-Rehner equation, the swelling degree decreases with the increase of cross-linking density and the worsening of polymer–solvent interactions [100–102]. A variety of stimuli that can induce the volume change of water inside the hydrogels can be applied, such as temperature, light, magnetic field, pH and ionic strength [103].

It is required for the soft robots to be operated in desired ways according to the specific tasks, and therefore, the deformations should be devisable and controllable. As is mentioned above, the deformations of the materials are based on two necessary factors, which are referred as materials and stimuli. Accordingly, the control of deformations is inseparable from these two aspects. For the homogenous materials exposed to homogenous stimuli, either in-plane deformations such as expansion and contraction when the volume of the material changes, or expansion in one direction and contraction in other directions when the volume remains constant, will occur. However, these deformations are usually not enough for the soft robots to perform complex tasks in the real-world applications, since more complexed deformations are essential, which can be achieved either by making the materials with heterogeneous structures or exerting the stimuli heterogeneously.

The controlled deformations can be obtained by endowing the deformable materials with heterogeneous structures at the micro- or macrolevel. On this occasion, the homogeneous stimulations are also enough for actuation of the soft robots. Since the deformability is based on the inherent property of materials, the control of material deformation is mainly reflected by the selection and adjustment of the factors that affect the deformability at the molecular level. SMPs are polymer networks comprised a fixed phase and reversible phase. The permanent shape of SMPs is determined by cross-linking. The transition shape can be obtained by processing polymer above the glass transition/melting point and then cooling it below a glass transition/crystallization temperature. The permanent shape can be recovered again once the material is heated. It is noticeable that fixation can also be accomplished by making use of any way that restricts the mobility of polymer chains and chain segments [82, 86, 104–110]. Liquid crystalline elastomers (LCEs) possess the properties of two states, including a crystalline solid and a liquid that makes them structural elastomers with anisotropic groups. The polymer chains are extended and self-organized under an applied external load in the liquid-crystalline phase, while chains are able to recover their coiled conformations after a transition to the isotropic phase. Any stimuli like temperature or light which can cause isotropization can be introduced for the phase transition [111–113].

The heterogeneous structures can also exist at the macrolevel, which means that the soft actuators can also be formed by the materials with different deformability combined as some specific ways to provide complexed deformations, and both the factors which can exert an influence to the deformability and the style of the structures should be taken into consideration. Various investigations have been carried out about the soft actuators based on different mechanisms with a diversity of structures. Soft actuators can be obtained by the integrations of materials with different deformabilities into bilayer structures. In particular, the bimorph structures comprised of two kinds of materials with different coefficient of thermal expansion (CTE) have been frequently studied attributing to their advantages of light weight, continuous shape change and actuation simplicity [114]. For instance, a soft jumping robot fabricated from the rolled carbon nanotube (CNT)/polymer bilayer composites was developed [115]. In this system, polydimethylsiloxane (PDMS) which was featured with biocompatibility, and physical and chemical stability, was selected to form one of the layers for the reason that it was a kind of elastomer with a large CTE. Accordingly, CNT was introduced as another layer to make the thermal stress in the interface formed with the asymmetric thermal expansion between PDMS layer and CNT layer, resulting to a rolling of the bilayer toward PDMS layer when the curing of PDMS was finished and the temperature decreased to room temperature. This soft robot with tubular shape was managed to be driven by electricity and sunlight to accomplish versatile biomimetic motions including ultra-large deformation from tubular to flat.

The similar mechanism can also be suitable for the fabrication of soft actuators with three-layered structures. A soft actuator was developed by introduce of a porous elastic cloth sandwiched between two kinds of materials, namely PDMS and polyimide (PI) tape, with different CTE [114]. By means of controlling the heat distribution, the designed motion can be implemented. Besides the structures with bilayer or three layers, materials with different deformability can also be combined into some special structures according to the application requirement.

The actuation of the soft robots can be realized by exerting the stimuli heterogeneously to homogeneous materials as well. Since the stimuli are provided by the equipment, design can be conducted in retrospect to the device setup or the way in which the stimuli are applied. One case in point was that the near-infrared (NIR) light irradiation could cause a gelatinization gradient across the thickness of a starch actuator (SA) film, leading to the gelatinization gradient of SA [116].

## Design Methodology of Endowing the Soft Robots with Artificial Intelligence

### Sensing

Humans are capable of feeling their circumstances and communicate with the other people. As a result, it is expected for the soft robots with AI to be able to interact with the environment and humans. In the aspect of feeling the circumstances, humans usually depend on their sense organs, such as skins, eyes, nose, ears and so on. Accordingly, intelligent soft robots acquire the perceptive abilities by being equipped with bionic skins, guiding systems, physicochemical sensors and so on. As to the communication with others, language systems can be made use of by humans to express their intention, while a variety of human–robot interaction interfaces have been developed for the soft robots to understand the users’ intentions.

As the primary mechanoreceptors of the human, the skin plays an important role to perceive various stimuli, such as tactile, temperature and humidity [117–119]. The research focused on the electronic skins (e-skins) that can emulate the skin of human body are of significant importance for the soft robots with AI [58, 120–131], which is inspiring and challenging. A variety of technologies have been developed for the fabrication of the tactile sensors and e-skins with precise and rapid sensing abilities, such as piezoresistive [58], capacitive [59], triboelectric technologies [60, 61] and so on. However, the sensors based on a single mechanism may suffer from the nonlinear friction and electrical disturbances, which limit their applications in case where precise sensing is needed [132].

**Fig. 8:**
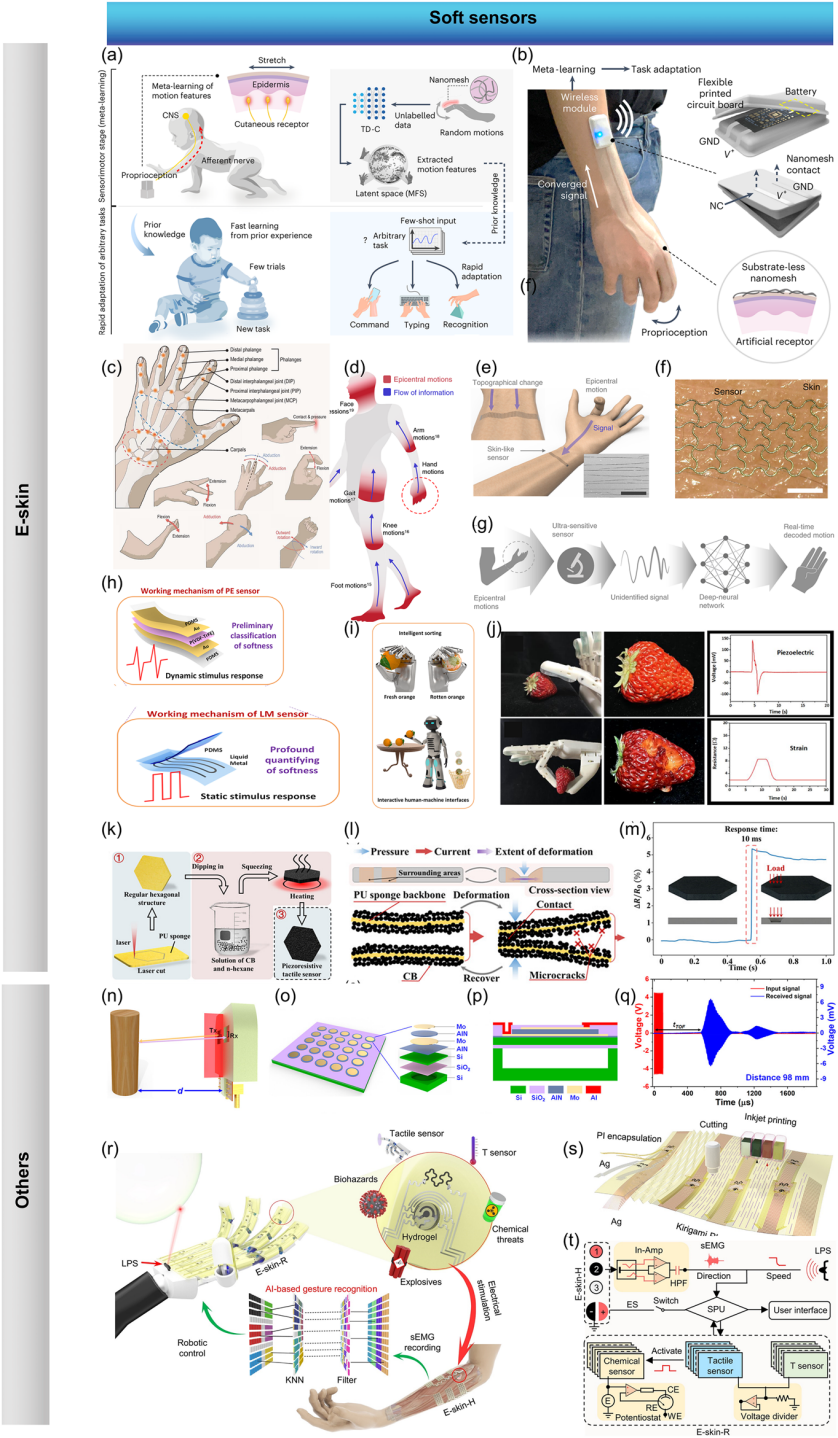
**a** Schematic illustration of meta-learning and rapid adaptation to new tasks for human. **b** Illustration for the formation of the artificial sensory intelligence system. **c** Graphical illustrations a variety of human hand motions. **d** Schematic illustration of the flow of information.** e** Schematic diagram of how to measure the epicentral motions of fingers. **f** Magnified image of the sensor on skin. **g** Illustration of the sensory system. **h** Structure and **i** the application of the e-skins with a piezoelectric layer and a strain layer. **j** Performance about the manipulator with single mold. **k** Preparation process of the piezoresistive tactile sensor composed of CB and PU. **l** Mechanism and **m** response time of the sensor. **n** Schematic diagram of how to measure the object at a distance. **o** Schematic of the pMUT array, and **p** the cross-sectional view of a pMUT unit. **q** Signals for the object with the distance of 98 mm. **r** Schematic diagram of the M-Bot. **s** Inkjet printing and automatic cutting used for the fabrication of e-skin-R.** t** Schematic signal flow diagram in the system of M-Bot

Some progress has been made recently in the field of soft sensors assisted by ML with excellent performance. One case in point was that a nanomesh receptor assisted with meta-learning was developed, which could learn from human cutaneous receptors by means of translating electrical resistance changes from fine skin stretches into proprioception [133]. The designed system was able to gather signal patterns from fine details of skin stretches for the extraction of proprioception information analogous to the way in which cutaneous receptors provided signal patterns for motion recognition (Fig. 8a). To be specific, the artificial intelligence system was formed by the nanomesh cutaneous receptors connected with a wireless Bluetooth module through a nanomesh connector (NC), which was then trained through few-shot meta-learning (Fig. 8b). It is noticeable that in real life, a diversity of hand motions can be realized by the fingers and wrists to accomplish fully interactions with the environment (Fig. 8c), and therefore the cognitive capabilities of humans to identify hand gestures have been simulated by a series of wearable sensors assisted with ML [134]. In addition to the recognition of hand motions, it is also essential to conduct the research of decoding the epicentral human motions for the reason that it can help to promote the motion tracking and health-monitoring (Fig. 8d). One case in point was that a single skin sensor was successfully developed to decode the epicentral human motions with the assistance from a DNN [135]. The schematic diagram of how to measure the epicentral motions of fingers was shown in Fig. 8e, and the magnified image of the sensor on skin was demonstrated in in Fig. 8f. To be specific, signals about the deformation of the wrist were detected which were then analyzed through the encoding network to simultaneously generate the current status of the motion (Fig. 8g). Besides the epicentral human motions, progress has also been made in the wearable skin biosignal sensors combined with ML, leading to the outstanding performance in various medical monitoring tasks [136].

Endeavor has been made to improve the sensitivity of the e-skins, and therefore the e-skins which based on multiple sensing mechanisms have been developed to percept sufficient information and respond to complex stimuli [60]. There are four types of mechanoreceptors on different regions of human skins to perceive static and dynamic mechanical stimuli [118, 119]. The two slow adapting (SA) mechanoreceptors [Merkel (MD) and Ruffini corpuscles (RC)] of fingertip skins can respond to low-frequency stimuli and produce a sustained signal to demonstrate the static properties of the stimulus [137, 138], while the two fast adapting (FA) mechanoreceptors [Meissner (MC) and Pacinian corpuscles (PC)] can recognize dynamic pressure or vibrations [139, 140]. Novel tactile sensors that could mimic the SA and FA mechanoreceptors simultaneously were developed by means of integrating a piezoresistive sensor for mimicking the SA mechanoreceptor and a triboelectric sensor for mimicking FA mechanoreceptor, for the reason that the triboelectric sensing layer could generate a pulse electrical signal only at the moment of contact and separation and the piezoresistive sensing layer was managed to produce a maintained electrical signal during sustained indentation [60]. The piezoresistive sensing layer composed of the CNT-coated textile and the Cu-coated textile electrode layer could sense the pressure for the reason that the contact area between these two layers changed with the variation of the applied external pressure, leading to the change of the current. The response time was measured to be as short as 40 ms, and the recovery time was ~ 20 ms. Even small changes induced by loading–unloading a plant leaf (70 mg) and a seed (180 mg) were able to be detected. After 1500 cycles of repeated loading–unloading of a pressure with 10 kPa, the relative current signals could still stay stable. Meanwhile, the triboelectric nanogenerator (TENG) sensing layer fabricated by assembling stitching structured core–shell (Teflon steel) yarns onto the cotton textile was able to sense different kinds of materials due to its inherent ability of losing/gaining electrons. Various kinds of materials, including polyethylene (PE), nylon, polylactic acid (PLA), Cu and natural latex which showed positive voltage signals and Kapton, polyvinyl chloride (PVC), polytetrafluoroethylene (PTFE) which showed negative voltage signals could be sensed. By making a comparison of the amplitude and polarity of the output voltage, the tactile senor was managed to identify the type of the contact materials, showing great potential in the field of automatic object sorting and separation during the recycling and fabrication processes. Furthermore, the surface morphology or texture could also be recognized by the TENG-based sensor which worked in a single electrode-based sliding mode. The abilities of material recognition and texture sensibility could be further enhanced by ML for the possible large-scale applications. In addition to the material differentiation and surface morphology recognition, the hardness of the object could also be sensed by the combination of the piezoresistive sensor and the TENG sensing layer.

Further to improve the sensitivity of the e-skins, the multifunctional e-skins, which can imitate the sensory capabilities of human skin to the largest extend, have become one of the goals to be pursued, since it can perceive various external stimuli, which is an essential ability for the interaction with the environment [60]. One way to realize the multisensory functionality is to integrate multiple sensors into a sensing array [50, 141–143]. In this case, the simple fabrication processes are ideal [144–146]. Another approach to achieve the multisensory functionality is to design one system with multiple stimuli responsiveness. In addition to the enhancement of the sensitivity and the enrichment of the functionality, another critical point is to solve the problems of the mutual interference. For instance, in a textile-based tactile sensors with both the piezoresistive sensor for mimicking the SA mechanoreceptor and a triboelectric sensor for mimicking FA mechanoreceptor, a medical textile tape was bonded on the top of the CNT-coated textile to ensure a conformal contact between the CNT-coated textile and the electrodes, which could avoid the mutual interference between these two sensing modes [60].

Moreover, the identification of the softness without permanent damage is another factor which should be taken into consideration when design the smart soft robotics. When unprejudiced contact force is applied to an object to determine the softness by the relationship between force and deformation, permanent damages may be caused. To overcome this shortcoming, a sensing system comprised of a piezoelectric layer to preliminarily classify softness assisted by ML and a strain layer to realize quantitative elastic coefficient recognition was developed [132], which was illustrated in Fig. 8h, i. The appropriate contact force was determined successfully according to the classification. Even some easily fragile objects like tofu and tomato could be grasped free of any damage owing to the combination of the softness classification and quantitative elastic coefficient recognition, which could not be realized by the manipulator with one sensing mode (Fig. 8j).

In addition to the components of the e-skins, elaborate designs can also be conducted on the structures of the e-skins. It was turned out that by the combination of the data augmentation method development and the sensor structure design, the deep learning (DL) algorithms’ generalization ability could be obviously improved. For instance, a tactile sensor for robot based on carbon black (CB) and polyurethane (PU) sponge was developed [147], as is illustrated in Fig. 8k. The change of the overall resistance was determined by the combined effect brought about by the microcracks and the backbones contact. Particularly, the small deformation led to the microcracks in the CB layers, which could decrease the conductance of local conductive paths, and therefore the overall resistance increased. When large deformation happened, the contact of the sponge backbones occurred, which could introduce new conductive paths, increasing the conductivity of the sensor. The detailed mechanism of the sensor was demonstrated in Fig. 8l. The hexagonal shape equipped the sensor with the ability of being combined seamlessly to cover large areas. Moreover, the regular hexagonal structure was also managed to facilitate a novel data augmentation method. A fast response time to a transient stimulus of as short as 10 ms could be achieved (Fig. 8m).

Besides the tactile detection, languages can also be recognized for the soft robots with AI. A piezoresistive sensing unit was designed for monitoring various physiological signals and joint motions, including for recognizing the specific words [60]. Particularly, the CNT-coated textile and the Cu-coated textile electrode layer were featured with porous structures and rough surfaces, and the contact area between the CNT-coated textile and the bottom electrodes changed with the change of the external pressure, leading to a current variation when a voltage was applied. As long as the sensors are attached to the throats of people, muscle movements could be detected when people were speaking. As a result, different words could then be recognized, and excellent repeatability in word distinguishment could be realized.

In addition to tactile sensing and word recognition, it is expected for the soft robots to be equipped with guiding systems so that appropriate positioning can be accessible [148–150]. The conventional guiding systems mainly include cameras and optical sensors [62]. Recently, some advanced guiding systems for the soft robots have been developed with the advantages of being more adaptable and low cost. For instance, a noncontact ultrasonic transducer that was power saving, simple and free of data complexity was fabricated to provide auto-positioning abilities for the soft robot [62]. The ultrasonic sensor was made up of two piezoelectric micromachined ultrasonic transducers (pMUTs), among which one served as an ultrasonic wave transmitter (T_x_) and the other was the receiver (R_x_). As was illustrated in Fig. 8n, when the transmitter was exposed to a specific signal, the pMUT diaphragm vibrated under the applied electric field due to the converse piezoelectric effect, leading to the generation of the ultrasonic wave from the transmitter. Once it touched the objects on the transmission path, it would be reflected, and be recorded by the receiver. The detailed composition of the pMUTs was shown in Fig. 8o, with the cross-sectional view illustrated in Fig. 8p. The transmitted and received signals for objects at different distances could be recorded, and for the object with the distance of 98 mm, these results were illustrated in Fig. 8q.

Besides the sensors for physical parameters, the chemical sensors for robotic platforms are also in high demand [18]. Since there are many toxic compounds that are threat to health and security, the detection and control of these compounds are essential. The soft robots are expected to be applied on the occasion where the amounts of hazardous compounds like pesticides which can make people fall into danger are needed to be traced, saving people to be exposed directly to the threat caused by the hazardous substances. This goal was accomplished by a multimodal robotic sensing system which was referred as M-Bot with the capacity of physicochemical sensing [18]. As was illustrated in Fig. 8r, this system included e-skin-R that was conformal with the robot, and e-skin-H that was conformal with the human skin. An inkjet printing technology with the key features of being scalable, rapid and cost-effective was available to fabricate the flexible sensor arrays with multiple perceptions, including the recognition of tactile, the sensing of a wide range of hazardous materials and the record of electrophysiology (Fig. 8s). In particular, silver served as interconnects and reference electrode, carbon worked as counter electrode and temperature sensor, PI for the encapsulation and target-selective nanoengineered sensing films as tactile sensor and biochemical sensing electrodes were serial printed. E-skin-H worked as the human–machine interface and was responsible for autonomous robotic control and object manipulation. Once the signals were acquired, a variety of ML were evaluated for the gestures to be recognized accurately. The procedures including the object approach, recognition, positive threat detection, tactile and alarm feedback could be successfully accomplished. The e-skin-R was managed to carry out the proximity sensing, the temperature and tactile perceptual mapping, real-time electrochemical on-site sampling and analysis of threat compounds, no matter they were in solution-phase or dry-phase. All the components mentioned above formed a closed-loop human–machine interactive system (Fig. 8t).

### Decision Making

**Fig. 9:**
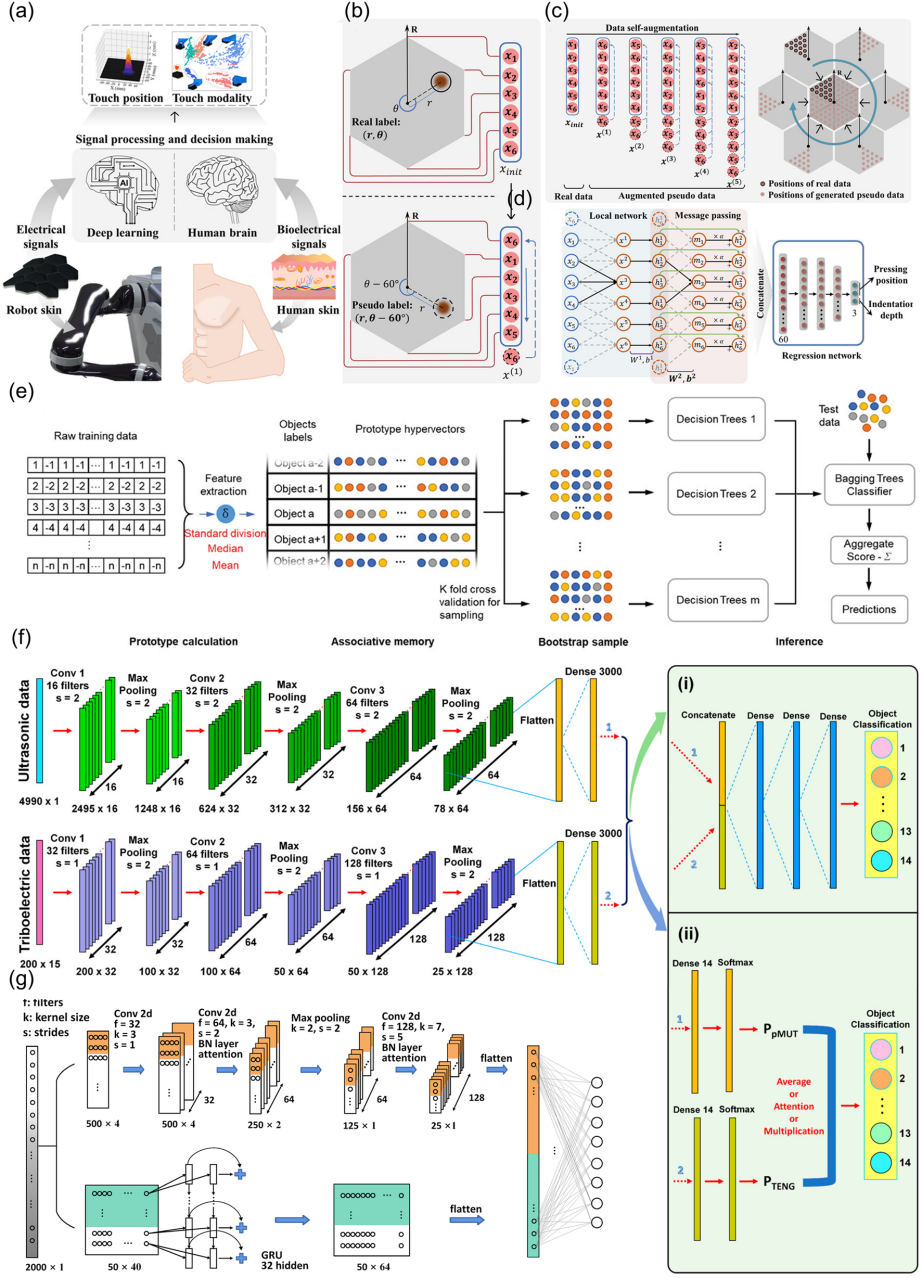
Illustration of **a** one step of data self-augmentation, **b** data self-augmentation, with a sixfold increase in initially collected data and **c** DNN architecture. **d** Schematics of how to construct the GRU-CNN. **e** Flow diagram of the machine learning process with the node-oriented decision tree algorithm selected. **f** Architecture of the network for the (i) feature-level and (ii) score-level fusion. **g** Process and parameters of the GRU-CNN

As for humans, once the information about the environment is received by the sense organs, the brains can process the signals and offer a clearer understanding of the surroundings for decision-making [151]. Just as humans can think, soft robots can be endowed with signal processing functions by ML (Fig. 9a).

DNN is one of the powerful techniques. However, it has its own disadvantages. Large amounts of data are to be collected when training a DNN model. To solve this problem, a combined design of both the sensor and the algorithms was conducted [147]. For instance, in an attempt to fabricate a tactile sensor for the human–robot interaction, the sensor with regular hexagonal structure prepared from CB and PU sponge was developed. A data self-augmentation method was then figured out. Because of the fact that the shape and position of the sample maintained the same when it rotated around its centroid at an angle which was a multiple of 60°, a polar system with real/pseudo-deformation could be established for the illustration of one step of data self-augmentation (Fig. 9b). By adopting this strategy, five times the initial training data of pseudo-data were obtained (Fig. 9c). A local network was then proposed, as could be seen in Fig. 9d. The data self-augmentation method was proved to be effective even when using only six pressing positions, and could reduce the data size by 88%.

As for an optimal ML training model, both the appropriate feature extraction method and optimized algorithm are indispensable. One case in point was that the node-oriented decision tree algorithm was chosen when ML was utilized to identify a variety of terrains for a soft crawling robot [23]. The reason why this algorithm was preferred was that it made it easier for the researchers to build multiple decision trees and take the average into consideration to fully exploit the advantages of various models. As could be seen from Fig. 9e, the features were extracted before the analysis, and the standard deviation, median and mean of the raw data were extracted as features. After classification, these features were packaged into several decision tree algorithms for training.

Besides, for the soft robots applied in the real world, it is common that there are several sensors containing useful features, and each of the sensors has its own limitations to provide the accurate information under any circumstances. It is ideal to take advantages of all these sensors synergistically to achieve the best performance. On this occasion, multimodal fusion is a promising efficient method to fully use complementary strength of different sensors. One case in point was the construction of the perception system for the soft robots comprised of an ultrasonic sensor and TENG sensors [62]. The neural network structure for multimodal data fusion was demonstrated in Fig. 9f. In order to deal with the mismatch in data format and dimensionality of the data from these two kinds of sensors, at the early stage, sectional CNNs were taken advantage of to handle the data obtained from the ultrasonic sensor and TENG sensors independently, after which a three-layer dense neural network was applied to fuse the multimodal data at the feature level.

The efforts have also been made to increase the training efficiency and enhance the interaction of features [132]. One point in case was that a two-stream convolutional network (gate recurrent unit [GRU]-convolutional neural network [CNN]) was developed to recognize the hardness of the objects to be grasped by the soft robots (Fig. 9g). The confusion map of models revealed that the CNN method was capable of assisting the manipulator to achieve above 94.27% accuracy, while features learned from the GRU-CNN model were managed to further improve the recognition accuracy to 98.95% even when the training data were limited.

### Actuation

The soft robots that possess AI can execute the tasks according to the environmental stimuli or human’s expectations. Various forms of energy can be converted into mechanical energy by the soft robots by means of tethered, untethered or biohybrid approaches [152, 153]. Tethered soft robots are usually available via change of fluidic pressure in hollow channels, electrically driven deformation or by passive deformation with the assistance of motors. Several excellent works have been carried out to develop the soft robots with great performance, such as fluidic driven soft robots [154], electro-thermal soft robots [155–157] and electro-thermal soft robots [158]. In contrast to tethered soft robots, the untethered soft robots are usually controlled by light, magnetic field, acoustics and chemical, which also show their advantages in many application scenarios [159]. For instance, the soft actuators can also respond to the products of chemical reactions [57].

Different designs have been carried out to make it possible for the soft robots to accomplish some specific and complex assignments [20, 160–166]. Sensors are always applied to assist the soft robots to complete the required motions, especially for those occasions where the accurate motion detection and tactile recognition are essential [167–171]. To get rid of the high nonlinear deformation and no-joint structure existing in the traditional sensors like potentiometers and encoders, it is important to fabricate sensors compatible for soft robots to achieve the desired effect. Additionally, the ML technique can be made use of to enhance the data interpretation and realize better manipulation. The excellent performance of the soft robots results from the combination of sensors and ML.

**Fig. 10:**
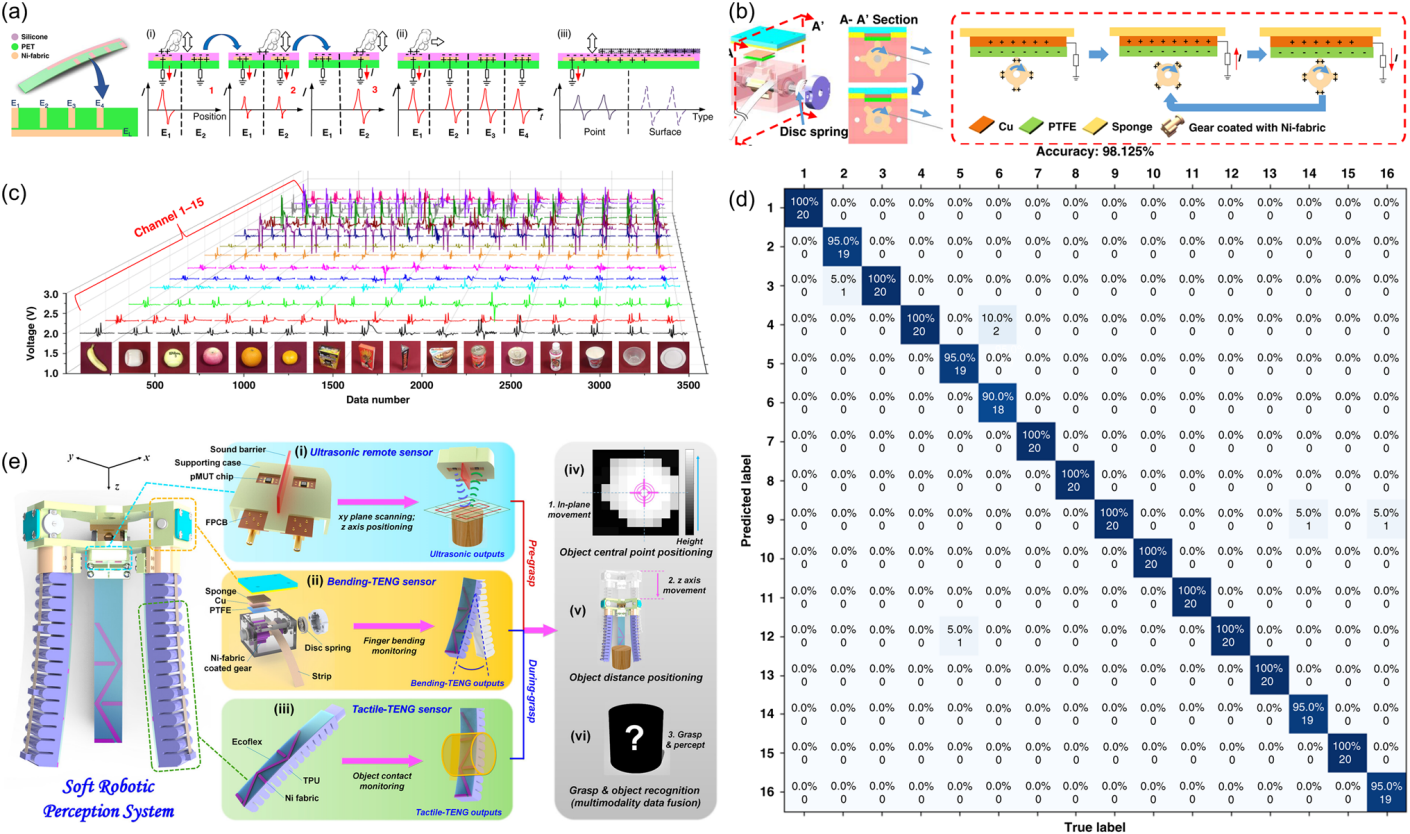
**a** Schematic diagram of the T-TENG sensor, and its working mechanism for (i) contact position detection, (ii) sliding detection, and (iii) contact mode recognition. **b** Schematic diagram of the L-TENG sensor, and its working mechanism.** c** Enhanced object recognition via machine learning, and **d** the confusion map for ML. **e** The soft robot equipped with the multimodal perception system

For example, the soft robotics trained by SVM algorithm which could capture the continuous motion and tactile information were designed [34]. Furthermore, the triboelectric sensory information was then trained by support vector machine (SVM) algorithm. To be specific, the patterned-electrode tactile TENG (T-TENG) sensor could provide the information of the sliding, contact position and gripping mode of the soft gripper. Five electrodes made of Ni-fabric were distributed on the polyethylene terephthalate (PET) substrate. The way of how the short and long electrodes were arranged was shown in Fig. 10a. The working mechanisms of this sensor for the contact position detection, sliding detection and contact mode recognition were demonstrated from Fig. 10a-(i) to a-(iii). As was shown in Fig. 10b, the length TENG (L-TENG) sensor was formed by a PTFE layer, which tended to attract electrons, and a gear covered with Ni-fabric with the Ni fabric layer tended to lose the electrons. The different stretching situations could be reflected by the performance of L-TENG sensor. In order to recognize more objects with different geometries, the three fingers of the soft actuator were all equipped with T-TENG and L-TENG sensors, providing fifteen channels of sensor output, and sixteen kinds of objects with different dimensions were used to be tested in the ML process. It was worthwhile mentioning the successful perception of the gripping status and the identification of the objects could be accomplished via ML, which demonstrated the great advantages of ML in the field of data interpretation. In particular, ML technology was proved to be an effective tool for dealing with classification problems, which was managed to deal with complicated input signals and extract their features from the dataset automatically via proper algorithms. As an effective supervised learning model, SVM, which was proposed to be applied in analyzing triboelectric output signals, was selected. The three-dimensional (3D) plots of the robotic sensor outputs are demonstrated as Fig. 10c. As could be seen in Fig. 10d, the recognition accuracy of trained model was able to reach 98.1%.

Another case in point was that a soft robotic system which was able to realize remote object positioning and a multimodal cognition with enhanced accuracy in object identification (∼100%) was proposed [62] (Fig. 10e). During the pre-grasp period, the robot was operated with in-plane movement to scan across, after which process, a topography map of the scanned area with various objects was obtained according to the reflected ultrasonic signals. The soft robot was then guided to the position where the objects lay via in-plane movement, and adjust the height through an out-of-plane movement to finally reach the suitable position. With the further assistant of the DL analytics, object identification with an enhanced accuracy (∼100%) was achieved. During the grasp period, the triboelectric bending and tactile sensors were made used of to get the specific information about the target objects, such as the shapes, materials and hardness. To be noticeable, multimodal fusion, which was proposed to be a promising approach for more robust sensory systems by utilizing the complementary effects of different sensors, was made use of. By taking advantage of the DL, object classifications were able to be accomplished. Thanks to the excellent object position-grasp-classify capability benefitted from the data fusion analytics, the intelligent soft robot could be qualified for various application.

## Design Philosophy Oriented from the Realistic Demand

### Smart Industry and Manufacturing

**Fig. 11:**
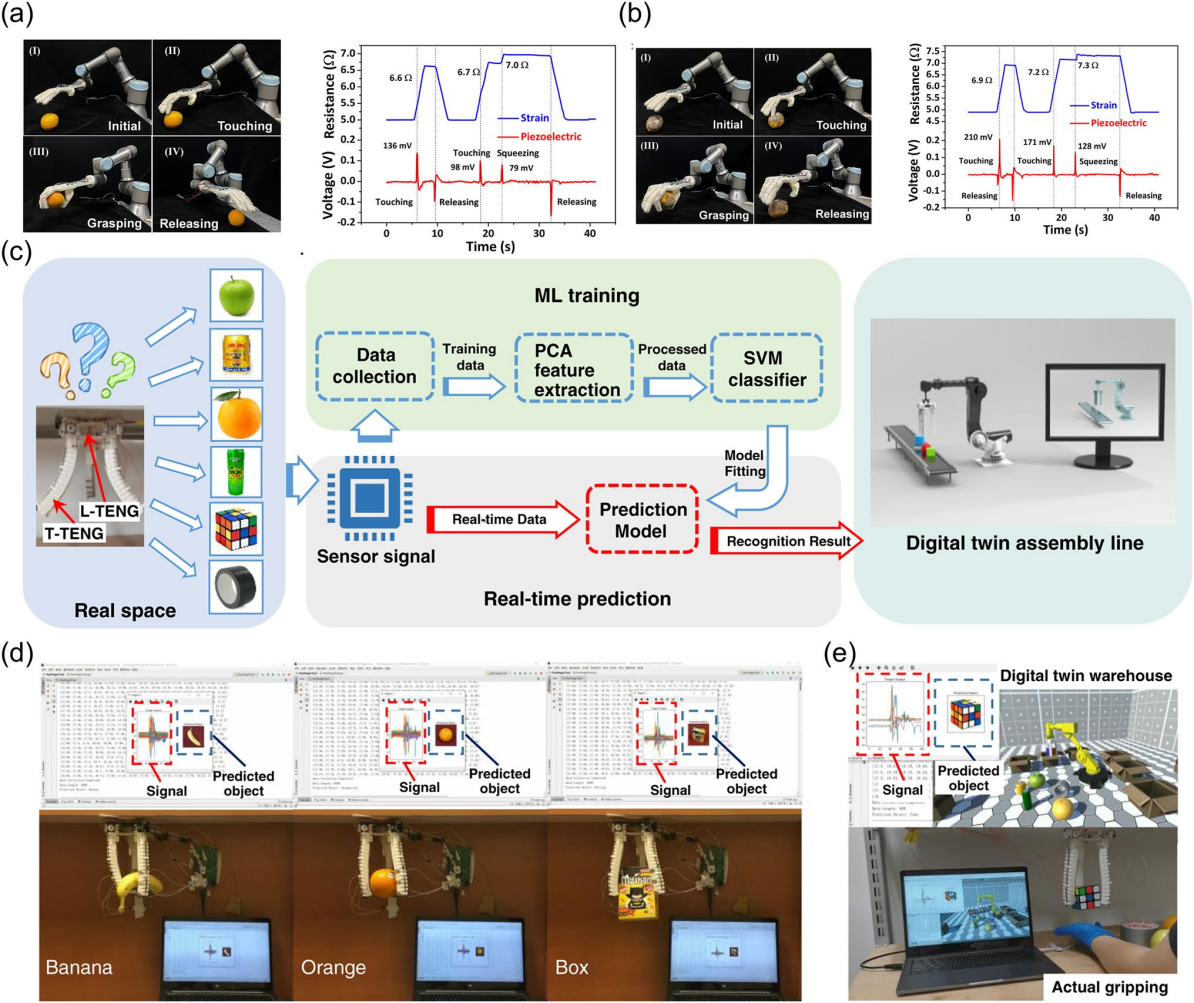
**a** Photographs of the process illustrating how the fresh orange was sorted by a manipulator, and the resistance and voltage output obtained from the sensors. **b** Photographs of the process illustrating how the rotten orange was sorted by a manipulator, and the corresponding signals from the sensors. **c** Flow diagram illustrating how the soft robot worked in digital twin system. **d** Object prediction effect provided by this system, and **e** the system interface for object recognition and digital twin warehouse application

In the future industry, soft robots with perception can take the place of people to accomplish more delicate tasks, which will make a difference to the whole industry. In addition to grasping objects, sorting is another common task in many fields, such as manufacturing, agriculture and so on. The smart sorting can be achieved by the soft robots with multifunctional sensing modules. As a prototype, the soft robot for the effective distinguish of the fresh and rotten oranges was fabricated [132]. By the integration of piezoelectric and strain sensors onto the manipulator, the fresh oranges could be distinguished from the rotten ones according to the different signals received from the sensors (Fig. 11a, b). The grasping force could then be decided based on the difference of softness between fresh oranges and rotten ones, making it possible to sort and grasp the delicate objects like oranges on the assembly line.

With the rapid development of 5G technology, great progress will be made in the field of smart manufacturing by using huge amounts of sensors under the IoTs framework to provide real-time sensory information [172–176]. For instance, a digital twin-based unmanned warehouse system was proposed by employing a soft-robotic sensory gripper [34], which was demonstrated in Fig. 11c. Both the automatic sorting and the real-time monitoring without cameras could be achievable by making use of this system. In particular, after trained with SVM, the object recognition was reliable. As was illustrated in Fig. 11d, not only the predicted objects, but also the real-time ones were clearly exhibited. When connected with a virtual space, the real-time recognition system could be successfully applied in the unmanned warehouse where a variety of objects to be gripped were randomly placed, as shown in Fig. 11e. In particular, the input signals collected by the sensors were used for the object recognition by the trained SVM model. The signals and the corresponding recognized results were all clearly displayed on the screen. After that, the virtual objects were sorted and placed into the proper boxes, while the same recognized objects were sorted in the real space.

### Intelligent Healthcare

The Coronavirus Disease 2019 (COVID-19) has highlighted the importance of adopting smart robotics to prevent infectious disease spread. The soft robots with AI can play an important role in noncontact collection of biosamples and nursing infectious disease patients [177]. Besides, the soft robots with AI can also leave positive effect on smart health care with a variety of health monitoring functions being provided [60]. Particularly, the soft miniature robots have the advantages of being flexible in size and the mobility to be operated in confined environments, making them promising candidates for the biomedical applications, including disease diagnosis, controlled release of drugs, minimally invasive medical treatments and so on [178–183]. It is noticeable that the magnetically controlled soft miniature robots are widely applied in the biomedical fields [184–186]. In addition, ML can propose constructive solutions to the difficulties faced up by the miniature robots used to execute the biomedical tasks [178]. When taking safety and efficiency into consideration, it is of high importance to equip these devices with reliable controllers. As for a magnetically controlled soft robot, it is necessary for the magnetic actuation system to offer enough force for the robot against flow rates, while the robust control framework should be managed to deal with uncertainty systems. When biofluids are not favorable, it is critical to provide the precise control and adapt to the dynamically changing environments. Moreover, the biomedical tasks put forward higher requirement for the robustness, accuracy and repeatability. As a result, high demands have been proposed for the soft robots that can be led to the target location precisely even in flow rate conditions [187–190]. Learning-based methods are able to provide constructive solutions for the fabrication of soft robots that can be suitable for the dynamically changing environments even in the cases where the existing analytical or numerical models are lack [191]. For instance, a broad learning system (BLS)-based visual servo control method was made use of to steer a microswimmer with the aim to track the desired trajectories [192]. Besides, deep reinforcement learning (DRL) has also been introduced to control problems, and the tests on the viability of using DRL-based approaches for magnetic robot control have been conducted [193]. A proof-of principle study based on DRL was conducted in which microrobots were operated in a simulated vascular network scenario by taking advantages of an actor-critic framework. The magnetic resonance navigation of a miniature robot was simulated with a Q-learning algorithm. Due to the dynamic environment, it is essential to introduce online estimation algorithms based on deep learning that are able to make use of any or all input and output measurements for updating the controller parameters into the adaptive control architecture.

A research focused on the soft magnetic miniature robot (SMMR) working in dynamical environments using a DRL-based control framework to accomplish the drug delivery and release tasks has been carried out [178]. A SMMR that could be actuated by a mobile electromagnetic actuation system integrated with three electromagnetic coils was fabricated. A simulator based on neural networks (NNs) was constructed, and a GRU-based soft critic-actor (SAC) algorithm was adopted, regarding the history state-action and the estimated flow rates as part of inputs. Taking the inaccuracy of the magnetic field into consideration, randomization technique was exploited, aiming at bridging the mismatch between the simulation model and real-world dynamics. It was proved that the performance of the framework was satisfied enough, and the physical SMMR could execute the goal-reaching and hovering tasks even with the existence of flow rates.

**Fig. 12:**
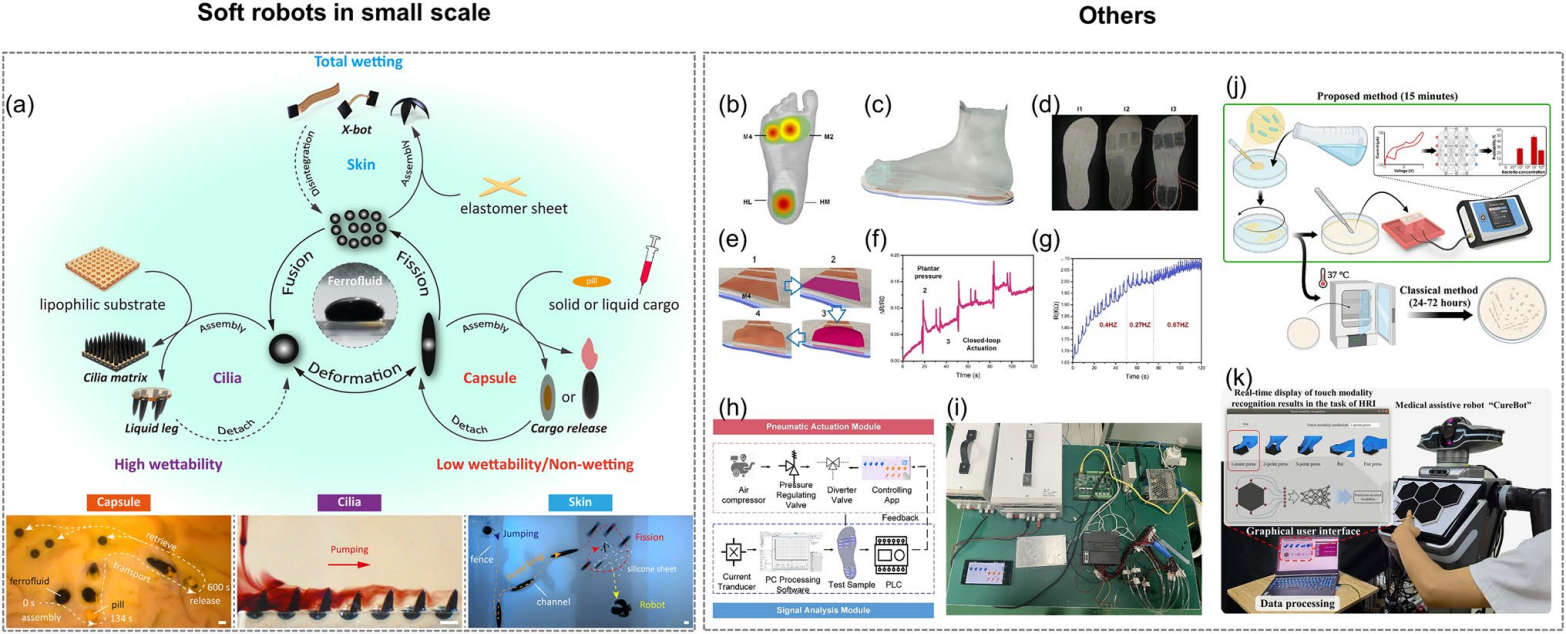
**a** Illustration for the multifunctionality of the wetting ferrofluid droplets. **b** Illustration for the partitioned plantar. **c** and **d** Different perspectives for demonstrating the therapeutic insole. **e** Schematic diagram for illustrating the working process of this active insole. **f** Closed-loop regulation, and **g** signal changes of patient walking at different frequencies. **h** Schematic diagram and **i** picture of the controlling system. **j** Brief scheme of electrochemical platform with the assistant of ML for bacteria detection. **k** Combination of tactile sensors with a large area on a medical assistive robot

Another case in point was that ferrofluidic wetting was exploited to control reconfigurability and fabricate miniature soft robots [240] (Fig. 12a). It was noticeable that multimodal motions could be realized, and the splitting and then re-fusing could also be achieved. The ferrofluid droplets were able to be configured in various forms, like a liquid capsule, a wireless omnidirectional liquid cilia matrix and a wireless liquid skin.

Soft robots can also play an important role in the pain relief caused by some common chronic complications. For instance, the diabetic patients are frequently suffered from diabetic foot, the symptoms of which are manifested in the abnormal distribution of plantar pressure. In an attempt to relieve this pain, a soft machine aiming at reducing the peak pressure of plantar pressure was developed, which was proved to be able to effectively improve the comfort of the diabetic patients [194]. As was shown in Fig. 12b, the plantar was partitioned into the metatarsal region (M2, M4) and the tarsal region (HL, HM) taking both the analyzing results of the plantar pressure distribution and the manufacturing versatility into consideration. The distribution of the functional composites and air chambers were arranged to point-to-point response to the abnormal stress. As was illustrated in Fig. 12c, d, there were three layers in the therapeutic insole, including a passive layer (l1), an actuating layer (l2) and a sensing layer (l3). The detailed procedures of how this therapeutic insole worked were demonstrated in Fig. 12e. In particular, the increase of the local plantar pressure could be detected by the sensing module, after which step the signal would be transmitted to the software and processed. After that, the actuation control command was then sent to adjust the local air pressure to make the chamber inflated at a certain magnitude, providing enough support for the patients. The periodic changes of the wearer’s plantar pressure could be detected, as shown in Fig. 12f. It was noticeable that the information of the signals from the patient’s walking at different frequencies with the therapeutic insole could also be gathered to verify the reliability of this active insole (Fig. 12g). Additionally, the schematic diagram and the photograph of the controlling system with a pneumatic actuation module and a signal analysis module were clearly shown in Fig. 12h, i.

DL, which is characterized by its ability of processing and analyzing large amounts of data, has also been employed to analyze a large number of biological images [195–199]. As is known, some lactic acid bacteria can make a positive influence on people’s health, such as curing gastrointestinal disorders, strengthening the host’s immune system and so on, which has already attracted the attention of researchers. Bacterial viability is usually deemed an essential indicator for optimal probiotic functionality, and the quantities of such lactic acid bacteria to enter the gastrointestinal tract alive should be enough. It is also important to control the number of viable bacteria during storage, for which purpose a reliable way to count the number of viable bacteria is always in high demand. In an attempt to detect the bacteria (Fig. 12j), a soft actuator comprised of hydrogel/eutectic gallium-indium alloy interface was developed combined with fast ML, in which case faster and cheaper bacteria quantitative analysis could be achievable [200]. The I−V characteristics at the interface between the eutectic gallium-indium alloy electrode and the hydrogel was applied to detect the concentrations of *S. thermophilus* and *B. Coagulans*. Such a device could keep the bacteria’ viability during detection period. It was proved that bacterial with the concentrations in the 10^4^ to 10^8^ cfu mL^−1^ range of the culture medium or in the dairy products could be accurately detected by the multilayer perceptron model.

In the healthcare scenarios, it is of significant importance for the soft robots to have the ability of intuitive recognition for the touch modalities, so that an effective interaction can be realized. Some progress has also been made in this field. For example, by the combination of several sensors with sixfold rotation symmetry, a large sensing area on a medical assistive robot, “CureBot,” was fabricated [147]. The different touch modalities could be recognized and displayed on the screen, which could further be associated with some control command (Fig. 12k).

## Design Strategies to Improve the Overall Performance of Soft Robots with Artificial Intelligence

### To Strengthen the Adaptive Ability

**Fig. 13:**
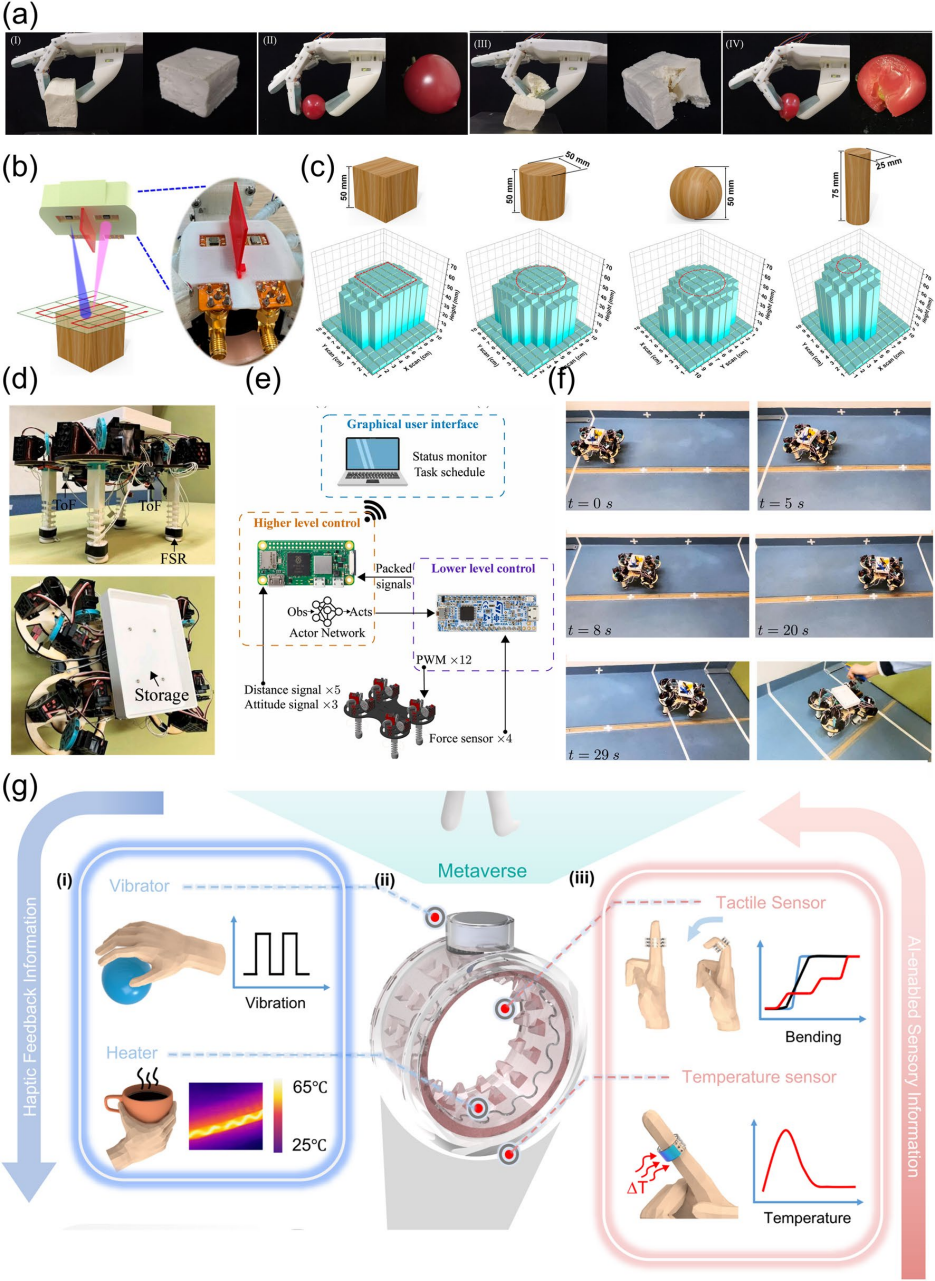
**a** Demonstration of grasping tofu and tomato using a manipulator. **b** Ultrasonic sensor integrated on a soft robot for object profile detection, and **c** its performance for different topographic profiles. **d** Photographs of the robot prototype, and **e** illustration of the control components. **f** Photographs to verify the robot to conduct transportation tasks. **g** Schematic diagram for the multimode haptic sensing and feedback

The rapid changing and diversified environment call for the intelligent robots with greater adaptability to the dynamic and various surroundings [16, 201–208]. Compared to the traditional robots that can only grasp the object with a fixed contact force, with the assistance of various sensors and ML, it is possible for the soft robots to grasp objects with proper forces according the hardness recognition [132]. Even fragile objects can be grasped by the soft robots without any damage (Fig. 13a).

In addition to the ability to adapt to different hardness, the soft robots are expected to be able to operate the randomly distributed objects to further enhance the ability to adapt to the environment. By introducing an ultrasonic sensor to the soft robot (Fig. 13b), the objects with different topographic profiles could be recognized [62], which was illustrated in Fig. 13c. Meanwhile, the ultrasonic sensor was also managed to locate target object.

It is also expected for the soft robots applied in the real world to meet the high demand of being adaptive to various terrains. A new design of quadruped robots was proposed for traversing complex terrains [24]. Different finite element and lumped parameter methods were made use of to fabricate the optimal gait controller for the soft-legged robot. Additionally, the optimal gaits were learned by means of the soft actor-critic methods and curriculum learning. To verify this effect, the learned gaits were implemented in an in-house robot as a prototype. As was illustrated in Fig. 13d, the robots were also integrated with abundant sensors, including four Time of Flight (ToF) sensors that were used to measure the surrounding distances and height of the robot body to the ground as well as four Force Sensing Resistors (FSRs) to recognize the contact forces between the feet and ground. The forces on the FSRs were reflected via the real-time electric resistances. The robot was manipulated via two-level control, which was demonstrated in Fig. 13e. The higher level made use of a Raspberry Pi Zero 2 W to communicate wirelessly. The PC acted as the transport for the MATLAB Simulink programs, while it also served as the Graphical User Interface (GUI), which demonstrated the real-time status and received tasks for the robots to be accomplished. The status information from the five ToF sensors and the lower-level controller was collected by the Raspberry Pi. The soft robot was managed to fulfil some transportation tasks, which was clearly illustrated in Fig. 13f. Firstly, some hand tools were selected as the payload to be transported, and two room dividers were placed at the front and back of the text field to reflect the light signals from the ToF and served as the localization references. It was expected that the robot was able to walk from the initial position to the front divider and stop at a specific distance. It was proved that the manually set yaw deviation could successfully be corrected, and finally the tools could be transported to the target position.

The enhancement of adaptive abilities is indispensable of the multimodal perception which can be learned from the multimodal sensing in the biological systems like human skin [209]. To be specific, tactile sensation is in close relationship with multidimensional factors, including macroroughness, fine roughness, slipperiness, hardness and temperature. The augmented tactile-perception and haptic feedback ring which was featured with multimodal sensing and feedback functions was developed [241] (Fig. 13g). The system was composed of TENG tactile sensors which were responsible for continuous bending sensing, flexible pyroelectric sensors which was intended for temperature detecting, eccentric rotating mass (ERM) vibrators to provide vibro-haptic feedback and nichrome (NiCr) metal wires for thermo-haptic feedback. Thanks to the multimodal sensing and feedback functions, an interactive metaverse platform with the features of cross space perception was successfully accomplished.

### To Save the Time Cost

**Fig. 14:**
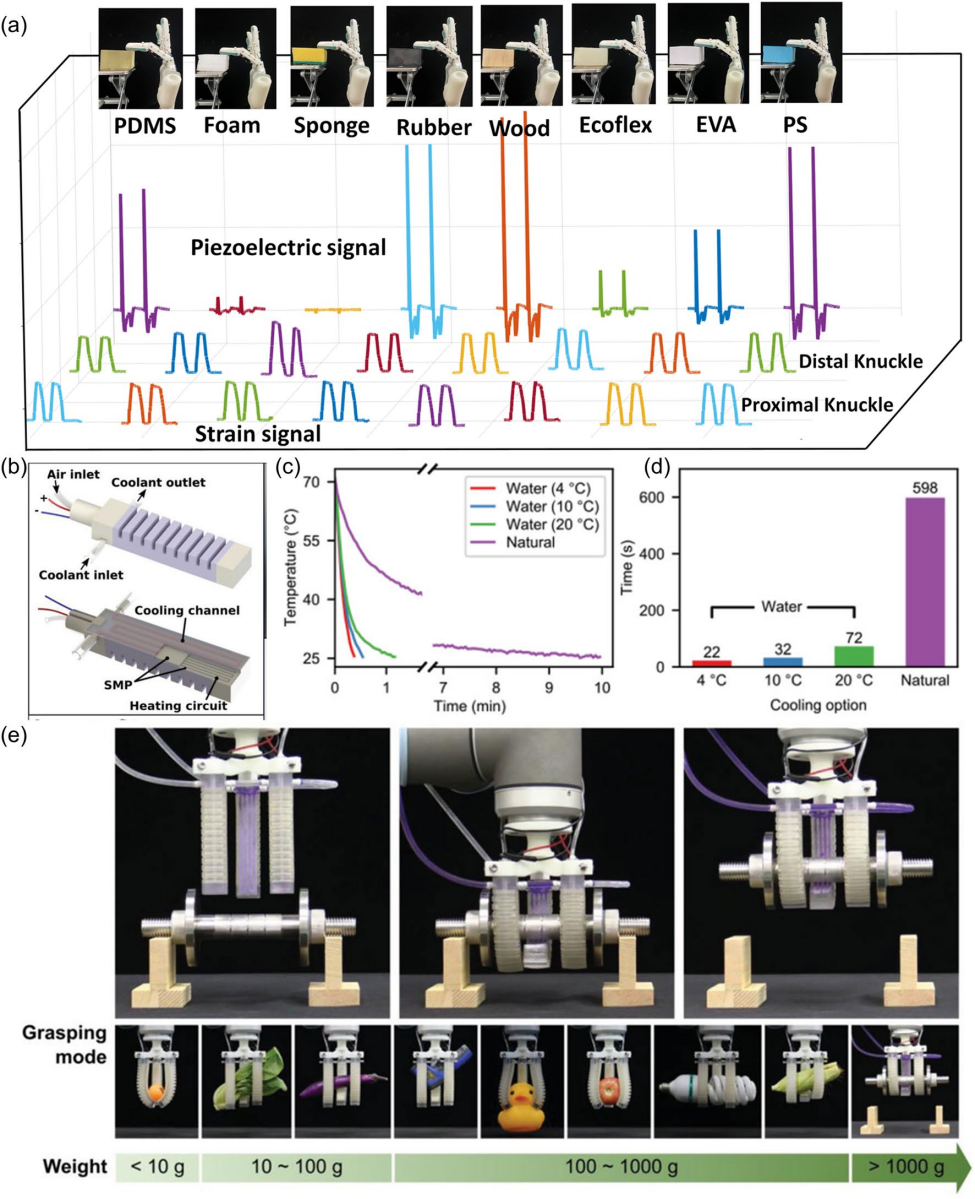
**a** Output comparison between the piezoelectric mode and strain mode. **b** Schematics illustration of the fast-response soft actuator. **c** Temperature–time relationship with water at different temperatures and natural condition. **d** Comparison of durations required to reduce the temperature. **e** Demonstration of the high load and shape adaptive ability

It is essential to save time and computational resources by selecting the sensory feedback properly [132]. For example, in a case of providing qualitative softness classification by ML, piezoelectric signal which varied obviously with the types of objects while ensuring the recognition accuracy was made use of to classify the softness (Fig. 14a). In addition to the sensory feedback, many other factors can also exert an influence on the computing time. For the soft robots with multi degrees of freedom (DOFs), a single sensor is not enough since it cannot distinguish the configurations of multiple deformations. For the purpose of solving this problem, cameras which are able to capture more signals have been applied. However, the use of cameras can lead to excessive computing time with many signals as redundancy. In an approach, a light-dependent resistors (LDRs) and light-emitting diodes (LEDs) were introduced to the soft robots instead of cameras or multiple sensors for the sensing and reconstruction of 3-D deformation [210]. It turned out that the accuracy of using four LDRs in one bellow was already as high as that of using four LDRs in every bellow for a soft continuum joint. Besides the soft continuum joint, a soft deformable membrane was also designed to ensure the feasibility of the proprioception method and the shape parameterization. In this case, a parameterization based on Bézier surfaces was conducted. Although the accuracy of the shape representation relied on the number of control points of the Bézier surface, the increase of the control point number would result in the more training samples, which was time-consuming. As a result, attention should be paid on the balance between the accuracy and the number of the control points.

In addition, it also takes a lot of time to learn a kinematic model for soft robots since the data of a large number of samples are required to be collected in a physical environment. To solve this problem, attempts have been made to generate the accurate dataset in the simulation environment and convert the model learned from simulation into physical reality by taking advantage of transfer learning. In one research, the performance of this learning-based method was evaluated by two hardware setups, among which one was soft robot with three chambers and another was soft manipulator [211]. Two models were used for the first hardware setup with the same Object350 Connex 3-D printer but slightly different materials, both of which could extend and bend in a 3-D task space. This kind of robot was actuated by an array of syringes with the assist of pressure sensors. Another setup was a soft manipulator comprised of three soft finger sections. Pressure was provided for each section. There were dual chambers in each finger so that symmetrical bending for both sides up to 120° could be achieved. The analytical forward kinematics (FKs) solution of soft robots in a virtual environment was computed by a geometry-oriented simulator. The computation time consumed by generating single sample point for the three-chamber robot and the finger manipulator was 4.3 and 1.2 s, respectively. To be time-saving, both the FKs and the Jacobian model were learned by the learning-based method in the offline stage. NNs were adopted to learn the functions in this research. The geometry-based simulator was selected for the reason that it was managed to further reduce the cost of data generation compared with the finite element method (FEM) and less physical data was need for the geometry-based simulator than for the analytical model to train the sim-to-real network. The sampling method in actuator space for the generation of training data points was proposed. The pressure range of each actuator was uniformly divided, ensuring that the distance between sample points was less than 1% of the workspace width.

The soft actuators are also to be able to response to the stimuli rapidly. For instance, a soft actuator featured with fast-response and stiffness-tunable capability was fabricated by hybrid multimaterial 3D printing [212]. Because of the printed Joule-heating circuit and fluidic cooling microchannel, fast heating and cooling rates could be realized (Fig. 14b). Temperature–time relationship with water at different temperatures and natural condition is illustrated in Fig. 14c, and comparison of durations required to reduce the temperature was shown in Fig. 14d. It turned out that heating from 25 to 70 °C could be accomplished within 10 s, while cooling from 70 to 25 °C could be achieved within 22 s. Both the high load and good shape adaptive ability could be provided by the soft gripper, which was demonstrated in Fig. 14e.

### To Enhance the Execution Effect

Multiple sensors are usually to be adopted for the soft robots in the real-world applications in order to fully percept their surroundings. In many occasions, the sensors and actuators are independent units. The emergence of self-sensing actuators that can percept their environment automatically makes the combination of sensing abilities and actuation into one element come true. For instance, a learning-based method to fabricate the soft robots with self-sensing properties was proposed. In this research, ML enabled self-sensing, so that no additional sensors were needed [213]. Specifically, the soft robots were usually composed of several actuators which were responsible for the complex movements and motions. When one actuator was moving independently, other actuators experienced a corresponding change, since they formed an interactively integrated entirety. This form of coordination could be learned to complete the self-sensing performance. It was turned out that in dual-chamber pneumatic actuators changes of internal pressures in both cavities could be detected when they were exposed to an external contact. These changes could be learned to offer a predication about the shape continuum of the actuator, as well as the magnitude of the external tip force which led to the disturbance. This system was developed for the assistant of wrist FE and RU deviation. The overall performance of the soft robots can also be enhanced by the development of the preparation technics [213]. For example, self-sensing actuators prepared through manual methods might result in inconsistent sensing, which would then induce unpredictability during their service period. In contrast, consistent sensing was managed to be achieved by 3D printing.

Besides, in order to improve the implementation effect, the entire system including the environment should be investigated as a complex but deterministic input–output system, rather than simply taking the soft robotic system into consideration [214]. One method that has been commonly used is to combine a model of the system dynamics with algorithms that can make a compensation for environmental disturbances [214]. This approach is more suitable for some traditional robots with their rigid body dynamics being easily modeled, and their structures usually perform limited degrees of freedom. However, soft robots are featured with nonlinear time-dependent physical–chemical properties and infinite numbers of degrees of freedom, making it difficult to model their body dynamics. Additionally, any changes in the environment can leave an effect on the overall system dynamics. For this purpose, an underwater soft robot was designed, and DNNs have been selected to explore the relationships between the inputs and outputs. The robotic platform was formed by two flapping mechanisms with curvature control powered by servomotors. Besides, a nonpermeable soft silicone skin was used for protecting the robot mechanisms in aquatic environments. An instrumented tank was applied to measure the force, in which the robot was fixed to a 3D printed clamp connected to a 6-axis load cell. The customized clamp was able to hold the robot along its body length, while it did not cause any interference to the fin motions of the soft robot. Additionally, the 6-axis force/torque sensor (Nano17) was attached to the clamp to measure the forces and torques generated during flapping. In this investigation, attempts were made to verify whether predications about a sequence of control inputs needed to precisely accomplish a sequence of target propulsive forces of this robot in a rapidly changing environment could be made or not. The soft robot and its environment were treated as a single system, which could then simplify the modeling process. It turned out that the actuation inputs to control this robot could be generated by means of DNN models.

**Fig. 15:**
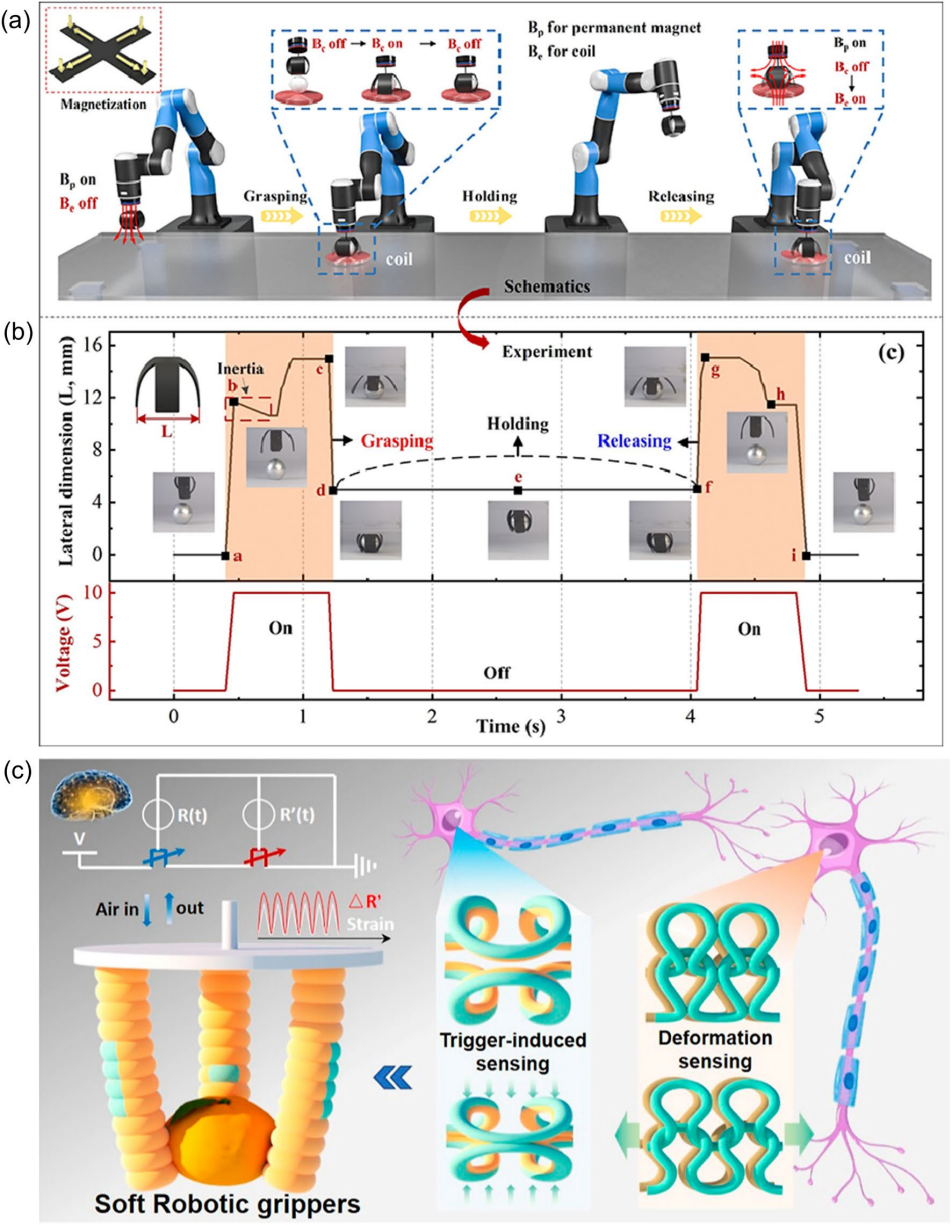
**a** Schematic diagram of the magnetic soft gripper, and **b** the change in the lateral dimension in the whole procedure. **c** Schematic diagram of knitted soft robotics with build-in textile-integrated multimodal sensors

The imbalance between flexible regulation and low energy operation usually limits the actuation effects of the existing systems relying on a single magnetic source. A novel hybrid-excited magnetic actuation approach was applied to fabricate the hybrid-excited magneto-responsive soft robots for manipulating objects [215] (Fig. 15a). With the static magnetic field to stabilize the deformation state of soft actuators, and the pulsed magnetic field to regulate the deformation, better actuation effects could be realized by the combination of the flexible regulation and low energy operation. Moreover, it was proved that the advantage of the system in energy consumption significantly increased since the holding-stage time increased (Fig. 15b).

In addition, the way in which the sensors are combined with actuators can also influence the actuation effect. The simple add-on strategies are commonly used currently, which can lead to some problems, like poor compatibility in mechanical properties and interfacing problems. In order to overcome this shortcoming, knitted soft robotics with build-in textile-integrated multimodal sensors were developed [239] (Fig. 15c). The knit structure acted as both an actuator and a sensor, which could then be integrated into knitted soft robotics.

## Design Consideration for Future Soft Robots with Artificial Intelligence

### To Be Fully Learned from Nature

The real-world application has put forward more abundant and higher standard demands for the soft robots. A lot of work has been carried out via fully learning from the living creatures which can endow the soft robotic systems with various advantages and make them more qualified for the real-world applications [216]. The behaviors of living creatures, ranging from land animals, aquatic animals, to aerial animals have been carefully observed, and researchers have come up with many ideas inspired by the outstanding features in actuation of these living creature. Efforts have been made from several levels, including the design of soft materials, the preparation of flexible electronics, the improvement of the fabrication method and so on [217].

**Fig. 16:**
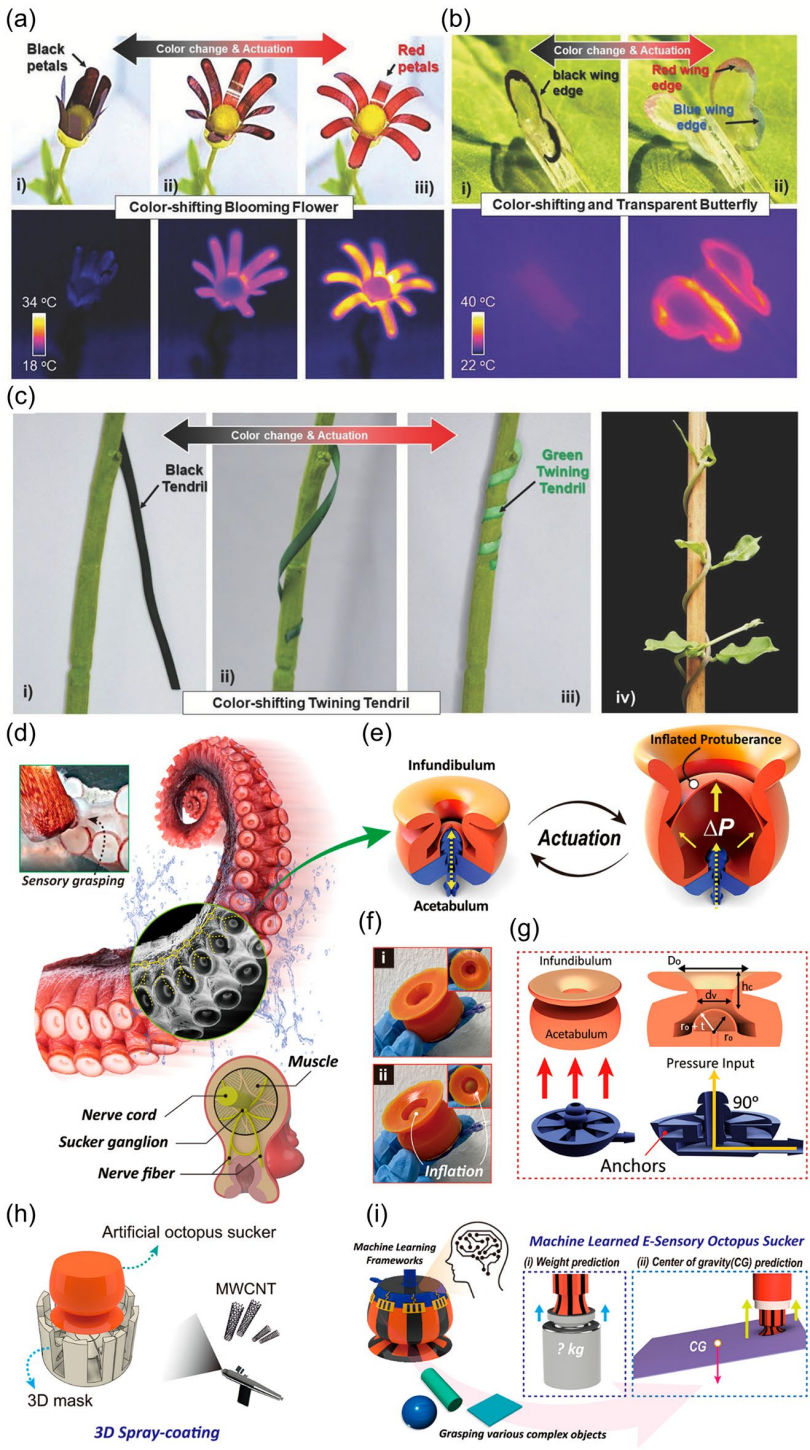
**a** Blooming and color-shifting of flower made from CASA. **b** Fluttering and color-shifting of butterfly wings fabricated from CASA with IR thermal images. **c** Twining and color-shifting of an artificial tendril. **d** The key features of octopus vulgaris arm. **e** Schematic illustration of an AOS during actuation. **f** Photograph of the AOS before and after actuation. **g** Schematic of the AOS with soft and rigid bodies. **h** Schematic of the AOS integrated with MWCNT strain sensors (E-AOS). **i** Schematic of the E-AOS assisted with machine learning

For instance, inspired by the creatures that could change their colors, biomimetic actuator with color changing ability was designed for soft robots, demonstrating a highly reversible behavior with very large deformation at considerably low temperature [218]. The color-shifting anisotropic soft actuator (CASA) was featured with a transparent bilayer structure formed by two films with different properties of thermal expansion, and the transparent silver nanowire (Ag NW) percolation network heater was also introduced into the system. A blooming flower (Fig. 16a), fluttering butterfly wings (Fig. 16b) and the twining artificial tendril (Fig. 16c) made from the CASA were demonstrated, which all showed color-shifting abilities with the temperature variation observed via IR images.

In addition to the abundant functionalities of the creatures in nature, inspiration from other aspects can also be obtained, such as their structures and switchable adhesion, and the performance can be further enhanced when introducing ML into the systems. In an attempt to learn from the switchable adhesion of octopus vulgaris arm (Fig. 16d), an electronically soft wet-adhesion actuator was developed [219]. There were some suckers dispersed on the arm of octopus. It was proposed that when a firm seal with the targeted object was established, the dome (protuberance) structure within the suction assisted to trap the water within the chamber, decreasing the internal pressure. Accordingly, the soft actuators with a versatile switchable adhesion even when they were under wet conditions were developed by learning from both the structure and attaching motion of the suckers of the octopus, which is shown in Fig. 16e and f. The artificial octopus sucker (AOS) was fabricated with a soft expandable part attaching to a rigid base fabricated in order to regulate the internal pressure (Fig. 16g). As shown in Fig. 16h, the strain sensors based on multiwall carbon nanotubes (MWCNTs) were introduced to be served as the E-sensory module, endowing the E-AOS with the ability to study the object (Fig. 16i).

In addition, both the proprioception and the exteroception are necessary for the soft robots to perform special assignments [23], including but not limited to working in a diversity of scenarios with various spaces and terrains, for the reason that accurate proprioception can collect the ontology information like the surface conformations, bending angle or curvature, and the exteroception enables the robots to perceive external stimuli [210]. Some factors can be mined in-depth to fully reflect the physical interactions between the soft robots and the ambient environment. For instance, the bending status of the soft robot’s limbs can cause the change in conformation, while it can also indicate the above-mentioned interactions.

In an attempt to achieve this goal, soft robots equipped with the shape-sensing electronic skin (SSES) which were able to reconstruct their shapes during movement were designed and fabricated [23]. The SSES was characterized with a mirror-symmetric structure with the conductive fabric served as common ground electrodes, and two piezoelectric sensing units that consisted of a polyvinylidene fluoride (PVDF) film sandwiched between two Ag electrodes, and the entire device was packaged by the silicon layers. The sensitivity could reach 0.146 V deg^−1^, demonstrating its potential applications for robots with high resolution. When integrated with the SSES, the soft robot was capable of sensing the surface conformations, which was indispensable for the acquisition of the real-time operating status. In particular, the curvatures at specific spots could be detected by the building blocks of the sensor array, after which they were analyzed and processed by computers to restore the conformation. The data collected by SSES could also be used to indicate the terrains crossed by the robot, since they could be reflected by the surface roughness. When ML was employed, it was expected for the soft robots to detect different environment which would be encountered by them in the real-world applications. Some new terrains were used to verify the generalization ability of the developed model.

### To Be Autonomously Operated

It is desirable for the components of the soft robots to be able to work without the consumption of the external power for the purpose of saving energy. To address this challenge, one approach is to design the systems with self-powered abilities, and some other method, like to make full use of the external stimuli, can also be adopted. In regard to the former method, a diversity of technology can be taken advantages of, including but not limited to triboelectric, piezoelectric and hygroscopic technology. The latter approach refers to those soft robots that can get the energy for their operation from the manipulated objects.

**Fig. 17:**
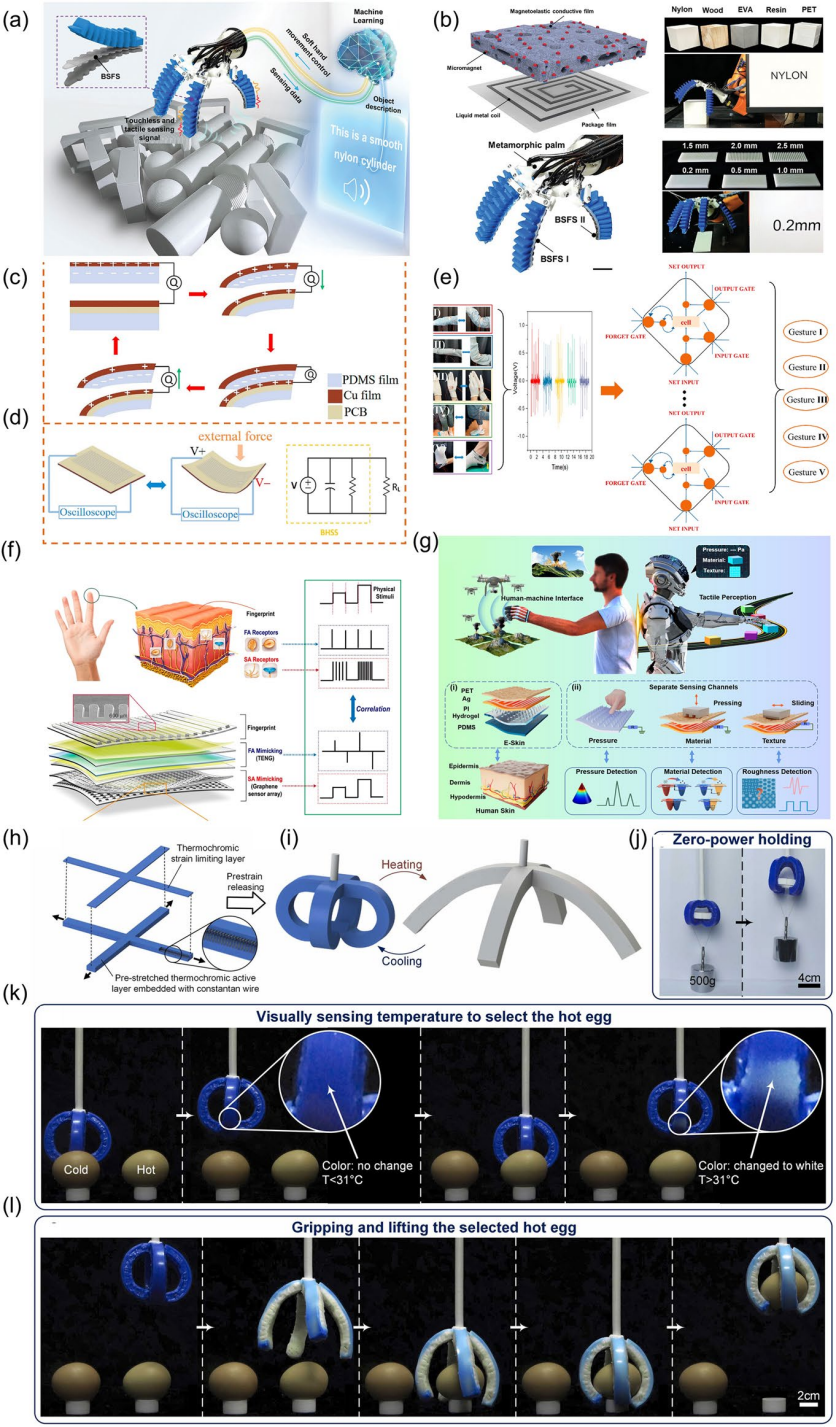
**a** Schematic illustration of the soft robot with a bimodal self-powered flexible sensor. **b** Structure of the sensor and the soft robotic hand integrated with the sensor to achieve material and surface roughness recognition. **c** Operating mechanism of BHSS composed of both piezoelectric and triboelectric units, and **d** the equivalent circuit diagram of the BHSS. **e** Gesture recognition assisted by the Long Short-Term Memory (LSTM). **f** Schematic illustration of the self-powered flexible neural tactile sensor. **g** Schematic illustration of the multifunctional e-skins working as human–machine interaction interfaces without external power source. **h** Schematic diagram of the soft gripper, and **i** the bending and the unfolding state of the gripper. **j** Illustration of how to lift a load of 500 g with zero power consumption. **k** Photograph of sensing the temperatures of the eggs for selecting the hot egg to be grasped, and **l** the progress of gripping and lifting the selected egg

Endeavors have been made to design the systems with self-powered features. As was shown in Fig. 17a, an intelligent soft robotic system assisted by a bi-modal self-powered flexible sensor to realize object description based on their physical properties was fabricated [220]. The self-powered sensor based on the TENG and giant magnetoelastic effect was comprised by a magnetoelastic conductive film and a packaged liquid metal coil, and was able to demonstrate a clear discrimination between touchless and tactile modes (Fig. 17b). By combining with ML, it was capable of describing materials and surface roughness. Some other attempts were made to fabricate the self-powered components with improved output for the soft robots [221]. For example, the biomimetic and flexible hybrid self-powered sensors (BHSS) formed by the integration of piezoelectrically actuated printed circuit board (PCB), nanofiber-based piezoelectric sensors and triboelectrically actuated Cu-triboelectric sensors were developed, and they were applied to an intelligent glove (Fig. 17c). The equivalent circuit diagram was shown in Fig. 17d. The hybrid self-powered sensors performed enhanced output with an open-circuit voltage (*V*_OC_) of 15 V and a short-circuit current (*I*_SC_) of 115 nA. As could be seen from Fig. 17e, the large-scale data gathered from the low cost and scalable BHSS sensor could be used by the LSTM architecture to realize dynamic gesture recognition. The fully mimicking the skin of a human finger could be achieved by the tactile sensors with self-powered abilities [30]. The sensors were formed by interlocked percolative graphene films to detect pressure via mimicking SA mechanoreceptors and TENG to detect high-frequency vibrations like FA mechanoreceptors, which could provide the output signals very similar to real neural spike signals in human skin (Fig. 17f).

In addition to e-skins, human–machine interaction interfaces can also be self-powered. Elaborately built multilayer structure was adopted for the self-powered human–machine interaction interface to realize the pressure detection and material type recognition with minimized mutual interference. For instance, a self-powered human–machine interaction interface to regulate unmanned aerial vehicle movements by monitoring human gestures was developed [222], which was shown in Fig. 17g. A biomimetic e-skin was primarily formed by a top layer of PET with nanoscale wrinkle patterns, an interdigital silver electrode that sandwiched between PET and PI, anti-dehydration and anti-freezing DN hydrogel electrode with micro-cone patterns, and PDMS served as the substrate. This e-skin had the separated sensing channels. In particular, the PET which was equal to epidermis was able to identify the type of the material based on the fact that the capabilities of various materials to gain or lose electrons during contact electrification were different. The interdigital silver electrode was made use of to mimic the inside mechanoreceptor to identify the material texture according to a stick–slip sensing mechanism. Based on the coupling of the triboelectric effect and electrostatic induction by using the microcone patterned hydrogel, the modest normal pressure could be detected without the consumption of the external power. By further combining it with a signal acquisition/process circuit, human–machine interaction interfaces can be obtained.

In addition to develop the self-powered soft robots, the energy from the manipulated objects can also be made used of for the soft robots to justify which one is the operation object and grip the qualified one with zero power consumption. For instance, a kind of thermochromic soft actuators based on liquid–vapor phase transitions was developed which could switch between the bending and the unfolding states with a mild temperature (31 °C), and the thermochromic microcapsules could equip the system with visually sensing ability [242] (Fig. 17h, i). As a proof of concept, the soft gripper with both sensing and actuation abilities was applied to grip a load of 500 g, which was 12.5 times of its own weight (Fig. 17j). Furthermore, when getting access to two eggs at different temperature, the soft gripper could judge which was the hot one and gripped it by bending in response to the heat from the egg. During the whole procedure, no additional power was needed, which was demonstrated in Fig. 17k, l.

### To Be Combined with Virtual Reality

The rapid advancements of AIoT have stimulate the development of smart soft robots for the digital-twin-based remote interactive applications, like robotic-assisted industrial automation and virtual shopping with immersed experiences [12]. The up-to-date status of the physical objects distributed sensory network, which include but not limit to the size, color, shapes, position and movement, can be detected and collected [12]. This cyber-physical system makes it possible for people to get advanced interaction virtually and remotely, so that real-time parallel control in unmanned working spaces can come true. Additionally, when introducing the augmented reality (AR) or virtual reality (VR) to the soft robots with AI, the robotic VR can make it possible for people to interact with robots in a combined manner, ranging from visual, auditory to haptic manner [177]. By adopting the remote interactive systems, people will enjoy better experience when they go shopping online, since online shopping has become one of the essential parts in our daily life [12]. The immersive experience will be enjoyed which is supported by both the information getting about the details about the products and the joint operations achieved by the soft robots.

**Fig. 18:**
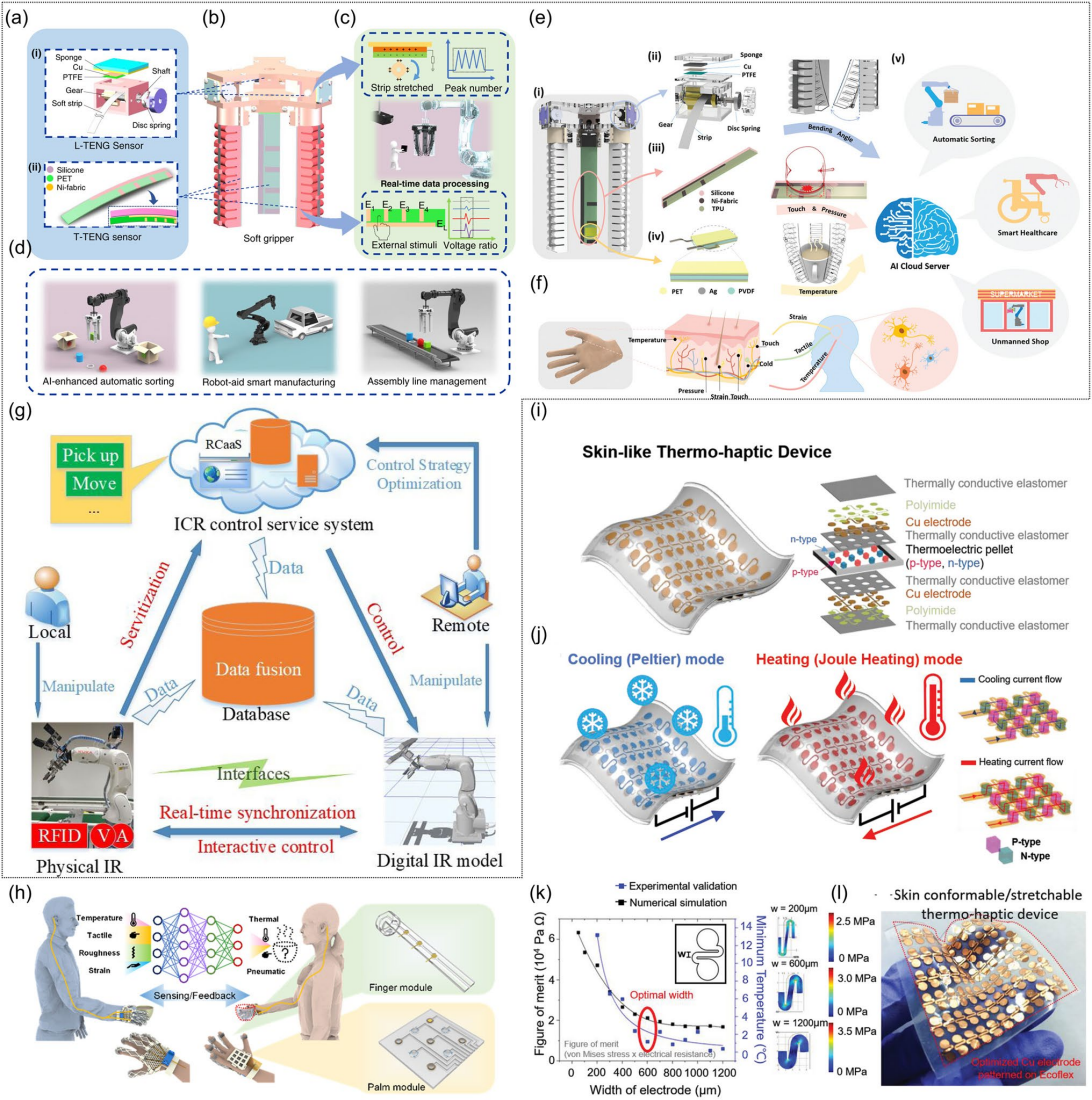
Construction of the soft gripper, including **a** TENG sensors with (i) L-TENG sensor and (ii) T-TENG sensor, **b** the soft gripper, and **c** the intelligent sensory data processing strategies. **d** Digital twin applications of this system enabled by the AI. **e** (i) The structure of the sensor-integrated smart soft robot. The structures and functionalities of (ii) L-TENG sensor, (iii) T-TENG sensor and iv) PVDF temperature sensor. (v) Various applications realized by this smart system. **f** Illustrations of how these corresponding functions are achieved by humans. **g** Framework of digital twin-based industrial cloud robotics. **h** Schematics of the intelligent soft glove for immersive communications. **i** Schematics illustration of the STH device, and **j** schematic illustration of the bi-functionality provided by this device. **k** Results of the numerical simulation and experimental validation for determining the optimum Cu serpentine electrode specification. **l** Photograph of the optimized serpentine Cu electrode on Ecoflex

A soft robot comprised of TENG sensors was fabricated for digital twin applications [34] (Fig. 18a–c). As was illustrated in Fig. 18a and b, the gear-based length sensor was responsible for the continuous detection of elongation, while the tactile sensor was managed to perceive the contact position and area. Combined with the signal readout strategies (Fig. 18c), the system could be applied in various digital twin areas, ranging from unmanned warehouse, smart manufacturing, to IoT, which was demonstrated in Fig. 18d.

There is an urgent demand for more interaction to provide better user experience, and the multifunctional perception systems contribute a lot to the better interaction. In another attempt to fabricate the soft robots to be used in the digital twin fields, a smart soft robotic manipulator with multifunctional sensors, including a T-TENG for contact position and area detecting, a L-TENG sensor for finger bending monitoring and a PVDF pyroelectric temperature sensor was designed [12] (Fig. 18e). As is illustrated in Fig. 18f, this multifunctional sensor was designed by mimicking human skin that possessed a diversity of receptors for detecting different physical information. When integrating the sensory information at the data level through the IoT module, the manipulator was managed to achieve more complex perception functions with the help of ML enhanced data analytic. Abundant information about the items, like tactile, deformation and temperature, could be perceived, so as to provide the actual status of objects in the real world.

It is noticeable that cloud computing resources have been proposed to be combined with robots in the intelligent manufacturing. A framework of digital twin-based industrial cloud robotics was proposed, which covered physical industrial robots (IR), digital industrial robots (IR), robotic control services and digital twin data [223] (Fig. 18g). It was worthwhile mentioning that digital twin made contribution to the realization of the bilateral mapping, real-time interaction and data fusion between digital robot models and physical robots. This framework was managed to synchronize and merge digital IRs and physical IRs effectively. Meanwhile, the bidirectional interaction between digital IRs and physical IRs made it possible for the fine sensing control of IRs.

AR and VR play an important role in many fields by providing immersive experience [224], which has also accelerated development of digital-twin technology. It is noticeable that the advanced functional materials and devices like soft haptic interfaces and soft sensors have promoted the development of VR and AR by offering soft and comfortable contact to human skin while conveying faithful feeling information. In addition to visual and auditory senses, some excellent work has been conducted focused on the tactile senses, especially thermal senses which reflected abundant information [225, 226].

Immersive communications can be accomplished with the assistance of intelligent soft robotics. The rapid advancement of flexible sensors [227–230] and machine learning [231–234] have provided the operators with the rich experience. In an attempt to achieve this goal, a modular soft glove with multimodal sensing abilities was proposed [235], which was illustrated in Fig. 18h. It was worthwhile noting that sufficient information including static contact, dynamic contact, vibration and strain could be detected just via a single design of basic structure without increased system complexity. With the aid of ML and IoT, augmented haptic feedback was realized, underlying its potential applications in the field of seamless communications among humans, machines and the virtual world.

Another case in point was that a skin-like thermo-haptic (STH) device with both cold and hot sensation a single structure was designed for the VR applications [236], the constitution of which was illustrated as Fig. 18i. It was worthwhile mentioning that p- and n-type bismuth telluride thermoelectric pellets were introduced into the system, which were mainly responsible for the rapid thermal response. This device a was managed to cool-down and heat-up the body surface by means of just switching the direction of electrical current (Fig. 18j). The numerical simulation and experimental validation for determining the optimum Cu serpentine electrode specification were carried out (Fig. 18k), and the photograph of the optimized serpentine Cu electrode was shown in Fig. 18l.

## Perspectives

Overall, the recent development, including but not limited to intelligence solutions to the sensing devices, ML and actuation systems of the soft robotics, is reviewed in depth. With the rapid development in the AI area, significant progress has also been made in the field of smart soft robots owing to their high-level safety, lower power consumption, less manufacturing costs and so on. The elaborate design lies in every aspect ranging from material selection of the components, structure consideration of the devices, to process optimization of the fabrication. Progress has also been made on preparing various kinds of sensors with more sensitive perception, proposing the algorithms and models with much higher accuracy, and developing the intelligent soft robots with much stronger execution ability, and the corresponding performance of these components are summarized from Tables 1, 2, 3. The comprehensive improvements have been accomplished by the design and fabrication the intelligent soft robotics with multisensory perception, close-loop human–machine interaction, ML capability and qualified execution for real-world applications, realizing their participation in the field of smart industry, intelligent healthcare and many other aspects where people can be substituted for some dangerous operations. There is a growing trend toward the fabrication of soft robots from biobased materials, the preparation of the sensors or actuators with self-powered features, and the combination of the AI with the VR or AR in the field of smart soft robots, which lead to more sustainable development and wider application of the intelligent robots. This review offers a keen insight into the design ideas for the advanced soft robotic systems with AI, and some perspectives for the smart soft robots applied in the future are proposed as follows:

1. The smart soft robots featured with excellent sensory capabilities are always in high demand. (a) It is desirable for the soft robots to mimic the sensory capabilities of human to a larger extend. For this purpose, the e-skins with more deliberate structures that can mimic the different types of mechanoreceptors of human skin have been developed to perceive both the static and dynamic mechanical stimuli. In the future, more investigations focused on the intelligent soft robotic systems with advanced sensors to percept sufficient information and respond to complex stimuli are expected to be carried out. (b) Taking the fact that the sensors based on a single sensing mechanism are susceptible to interference from environmental factors, multifunctional sensing schemes need to be put forward, which can also equip the soft robots with the capability of interacting with the surrounding environment precisely, rapidly and safely. Sufficient information is required to be sensed so as to connect the robots with their surrounding environment adequately. Great progress has been made in the development of tactile sensors and e-skins, and recently some chemical sensors have also been integrated onto the intelligent soft robots for the in-situ threat compound detection with the sensitivity of 0.95 A cm^−2^ ppm^−1^. The research which is focused on the accomplishment of a full imitation of human sensation, neuronal transmission and adaptive motility by the intelligent soft robots is inspiring. In the future, more advanced designs concerning about the other perceptions, like visual sense and auditory sense, can be carried out. Besides, in addition to the sense of pressure/force, tactile sensors which can accomplish more complex object recognition are desirable. Multiple sensor arrays that can offer more abundant information are integrated onto the devices. It is noticeable that in these cases mutual interference in multiple stimuli perceptions needs to be overcome. (c) More critical insights into the smart soft robots with both proprioception and exteroception are expected in the future work. Many attempts have been made on perceiving external stimuli (exteroception). In contrast, explorations concerning the smart soft robots with proprioception, which is also of great importance for recognizing the surface conformations and augmenting the ability of soft robots to reconstruct their shapes, are not enough. (d) Efforts also need to be made on enhancing the spatial resolution of the sensors especially for those occasions where various physical stimuli are exerted at the same position of the sensors on the soft robots.
2. There is an urgent demand for the intelligent soft robots that can exert positive impact on our ecosystem. (a) More sustainable materials are expected to be developed and exploited for the fabrication of intelligent soft robotics without sacrificing their overall performance. The exploration about applying the biodegradable materials in both the fabrication of the soft actuators and the electronic components should be made. On the one hand, the reversible actuation under cyclic stretching should be guaranteed when biodegradable materials are used. Ecoflex, which has been frequently applied to make the actuators for the soft robots, is a biodegradable aliphatic–aromatic co-polyester, although it is completely fossil-based. It turns out that cellulose and starch are sustainable materials with faster degradation rates when compared to synthetic bioplastics, and some attempts have been made to prepare soft actuators from the starch. In the future, more bioderived monomers instead of fossil-based ones can be made use of for the fabrication of soft robotics. One the other hand, it is ideal that the electronic components for the intelligent soft robots are biodegradable. To achieve the interaction with the environment, various electronics are introduced into the sensor networks, control systems or feedback modules of the smart soft robots. All paper-based piezoresistive pressure sensor has been made with good stability and high sensitivity. More factors concerning about the environmental problems can be taken into consideration when designing these electronic components. (b) More self-powered approaches are expected to be taken during the fabrication of the intelligent soft robots. The sensing mechanisms based on nanogenerators, like piezoelectricity, triboelectricity and pyroelectricity, are ideal selections that can achieve low power or even self-powered perception. Triboelectricity, and piezoelectricity for stimulus detection, and pyroelectricity and thermoelectricity for temperature perception have been taken advantage of to form the sensor networks for the soft robots, in which cases electrical signals can be generated by the sensors themselves instead of external electrical bias. In addition to self-powered sensors, the research on soft actuators for the robots that can be driven without artificial energy supply and intervention is also challenging. The key technology lies in many aspects, including how to harness the natural energy and how to respond rapidly. Efforts have been made to make the self-locomotive soft actuator stimulated by natural sunlight. It is expected for these self-locomotive soft actuators to be equipped with ML to show more brilliant performance in interaction with the surrounding environment in the future.
3. The explorations focused on the intelligent soft robots actuated by a diversity of method can be made. Soft actuators can convert multiple energy inputs into mechanical energy outputs to assist the soft robots to accomplish complicated work. Tethered synthetic soft actuations are featured with large force output and agile deformation, while the untethered soft actuators also have their own advantages. For instance, light can be modulated with both high temporal and high spatial resolution by making use of the developed equipment. As a result, both wavelength-selective and wide-spectrum light can be offered by different devices to actuate the soft robots according to the optical absorptive character of the systems. Furthermore, many environmental stimuli, like humidity, and chemical substances can also be made use of for the actuation of soft robotics. Much efforts have been made to fabricate the intelligent soft robots with pneumatic actuators, making them successfully be applied in the real-world scenarios, including but not limited to smart industry and digital twin applications. Besides, magnetically actuated soft robots combined with machine learning have also find their potential applications in biomedical fields, such as minimally invasive medical treatments, disease diagnosis and so on. Endeavors have also been made to deal with the dynamically changing environments and realize precise control of the soft magnetic miniature robots by taking advantages of the DRL method in real-world scenarios with the presence of flow rates. In contrast, the investigations concerning about the intelligent soft robots actuated by other method are not enough. Future work can be focused on the intelligent soft robotic systems actuated by more abundant stimuli to accomplish specific tasks.
4. Effort can be made on the development of the flexible and wearable components in the soft robot systems, since the soft robots are widely applied in the wearable applications, such as retracting garments, devices for haptics, neuroprosthetic hand and so on. To endow the systems with excellent air permeability and comfort feeling, more and more textiles which are mechanically robust, breathable and soft have been introduced into this field. Furthermore, efforts have also been made to solve the problems in the aspects of surface and interface integrations. When using the cotton fabric as the substrate materials, the as-prepared sensors can easily conform to different body parts or complex surfaces of soft robotics. The flexible sensors that can realize softness recognition of touched objects have also been developed. In addition to the sensors, flexible and wearable abilities are also ideal features for the HMIs, which can endow the systems with wearability and comfortability.

**Table 1:** Summary of the state-of-the-art soft perception systems for the soft robots with artificial intelligence

		System	Response time	Features	Being multifunctional	Refs.
Exteroception	Tactile perception	Ecoflex & PTFE-based triboelectric pressure & bending sensor	8 ms	Being self-powered	Yes	[62]
		Triboelectric tactile sensor	–	Being self-powered	No	[34]
		Carbon black-coated polyurethane sponge piezoresistive sensor	10 ms	–	No	[147]
		A piezoresistive sensor and a triboelectric sensor	40 ms	Partially being self-powered	No	[60]
		A T-TENG and L-TENG sensor	-	Yes	Yes	[12]
		A piezoelectric sensor and strain sensor	12 ms for piezoelectric sensor and 163 ms for strain sensor	Partially being self-powered	No	[132]
		Piezoresistive pressure sensor based on a printed silver nanowires (AgNWs)/nanotextured PDMS (N-PDMS) sensing film	–	–	Yes	[18]
		Skin-inspired multilayer microstructure to sense contact pressure and temperature	–	–	Yes	[50]
		Flexible neural tactile sensor	< 3 ms	Being self-powered	No	[30]
		Hybridizing both Cu-biomimetic PDMS triboelectric sensors	–	Being self-powered	No	[221]
		A bimodal sensor (BSFS) based on the TENG and giant magnetoelastic effect	10 ms	Being self-powered	No	[220]
		A triboelectric pressure sensor	10 ms	Being self-powered	No	[222]
	Temperature sensitivity	Pyroelectric sensor	–	With sensitivity of 1 °C and temperature range of 0–50 °C	Yes	[12]
		Hydrogel sensors	–	Temperature coefficient of resistance (TCR): −3.76% °C^−1^ and temperature range of 25–75 °C	No	[237]
		Printed carbon-based material	–	With a temperature range of 27–45 °C	Yes	[18]
		Concentric annular chrome/platinum (Cr/Pt) thin films	–	With sensitivity of 0.0016 °C^−1^	Yes	[50]
	Visual navigation	Ultrasonic auto-positioning system	2 ms	With a resolution of 10 mm and low power consumption	Yes	[62]
		Two flexible optical waveguide sensors	–	Anti-electromagnetic interference	No	[51]
	Chemical or bacteria detection	Biochemical sensing electrodes	–	With the sensitivity of 0.95 μA cm^−2^ ppm^−1^ and the detection limit of 10.0 ppm	Yes	[18]
		Soft hydrogel/ eutectic gallium-indium alloy	–	With the detection limit of 10^4^ to 10^8^ cfu mL^−1^	No	[200]
Proprioception	Shape reconstruction	Optical sensors	–	To be low-cost	No	[210]
		A SSES based on a differential piezoelectric matrix	36 ms	With the precision of 0.0025°	Yes	[23]

**Table 2:** Summary of the machine learning for the soft robots with artificial intelligence

Aim	Algorithm	Input	ML-enabled function	Accuracy	Refs.
Robotic manipulator	Data fusion algorithms	Multimodal data	Object recognition	∼100%	[62]
Soft gripper	SVM	Triboelectric sensory	Object identification	98.1%	[34]
Medical assistive robot “CureBot”	DL-based algorithm	The loading pressure and indentation depth	Recognizing five touch modalities	94.62%	[147]
Soft gripper	ANN	The voltage signals obtained from the TENG sensors	Perception of surface textures and material types	99.98%	[60]
Soft manipulator	1D-CNN ML algorithm	The voltage signals obtained from the T-TENG and L-TENG sensor	Recognition of the grasped objects	97.143%	[12]
Robotic manipulator	GRU- CNN	Piezoelectric signal	Softness classification	98.95%	[132]
Robotic joint and a deformable membrane	LSTM network, an FNN, a support vector regression (SVR), and a multivariate linear regression (MVLR)	Signals from the LDRs and LEDs	Conversion of the captured signals into shape parameters of soft robots		[210]
Soft Actuators	Adam algorithm	Data from strain and temperature sensor	Prediction of the soft actuator’s body posture changes and identification contact events with or without thermal stimuli	86.3%	[237]
Multimodal robotic sensing system (M-Bot)	kNNs	The surface electromyography signals collected from the human body	Remote robotic control and in situ threat compound detection	97.29%	[18]
An underwater bio-inspired soft robot	DNNs (multilayer perceptron (MLP) and LSTM)	Measured force and torque	To accomplish rapid modeling of bio-inspired propulsion	–	[214]
Pneumatic soft actuator	Adam algorithm (LSTM)	Pressure readings in the two pneumatic chambers	Prediction of bending angle and contact force	–	[213]
Bio-electrochemical platform	Multilayer perceptron algorithm	Current–voltage data	Bacteria detection	94%	[200]
Soft robot	Node-oriented decision tree algorithm	Voltage signals from shape-sensing electronic skin	Identification of various terrains	98.2%	[23]
Soft magnetic miniature robots	GRU-DRL algorithm	History state-action and estimated flow rates	Goal-reaching and hovering tasks for a soft robot	–	[178]
A soft quadruped robot	Reinforcement Learning (RL) (Soft Actor-Critic (SAC) method)	Gait of the quadruped robot	Optimal gait control	–	[24]
Soft machines like cardiac-mimicking actuator and active therapeutic insoles	–	Resistance signals	To get information on the stiffness, deformation and pressure of the material at different temperatures	–	[194]
Soft robot	NNs	Samples in a simulation environment	To solve the inverse kinematic (IK) problem on soft robots with highly nonlinear deformation	–	[211]
A robot hand	NNs and DL	Sensing data from quadruple sensors, including pressure sensing and temperature sensing	Recognition of different shapes, sizes, and materials in a diverse set of objects	94%	[50]
Soft optoelectronic sensory foams	kNN	Deformation mode and the values of angle	Detection of the robot own deformation and prediction of the magnitude of the deformation type	∼100%	[53]
A soft robot	NNs	Signals from the SA and FA mimicking sensors	Classifying fabrics possessing complex patterns	99.1%	[30]
An intelligent glove	LSTM	Signals from pressure sensors	Gesture recognition	82.3%	[221]
Soft fingers	CNN	Signals from bimodal flexible sensor	Description objects based on their physical properties, including materials, surface roughness, and shapes	97%	[220]
A wearable drone control system	CNN	Signals from hydrogel-based electronic skin	Material and roughness identification	Identifying materials 95.00% and textures 97.20%	[222]
Soft Actuator for underwater teleoperation	kNNs and light gradient boosting machine (LightGBM)	Signals from optical waveguide sensors	Recognition of 12 contact positions	99.82%	[51]
Knitted sensing textile	Random forest classifier	Signals from the TENG	Posture recognition	96.6%	[238]

**Table 3:** Summary of the output performance of the actuation systems for the soft robots with artificial intelligence

Actuation method	Material	Reversibility	Remote controllability	Additional functions	Application	Refs.
Pneumatic actuation	The filament material of thermoplastic polyurethanes (TPU)	Yes	No	With triboelectric pressure & bending sensor, ML-enabled object recognition	Automatic sorting, unmanned shops, and healthcare assistance	[62]
Pneumatic actuation	TPU filament	Yes	No	With triboelectric tactile sensor and length sensor, ML-enabled object identification	Virtual assembly lines and unmanned warehouse applications	[34]
Pneumatic actuation	TPU filament	Yes	No	With a T-TENG, L-TENG sensor, and a PVDF pyroelectric temperature sensor, ML-enabled recognition of the grasped objects	HMI for unmanned working space	[12]
Pneumatic actuation	Black PLA filaments	Yes	No	With multiple sensors and ML to fuse the captured signals into shape parameters	Soft robot with reconstructed 3-D shape	[210]
Pneumatic actuation	EcoFlex	Yes	No	With strain and temperature sensors, and ML to predict the soft actuator’s body posture changes and discern contact events with or without thermal stimuli	Soft robot for multimodal sensing discrimination	[237]
Pneumatic actuation	Eco-flex	Yes	No	With ML assisted electronic skins for proprioception and exteroception	Soft robot for real-world tasks with the ability of identifying various terrains	[23]
Magnetic control	Ecoflex	Yes	Yes	With ML assisted goal-reaching and hovering in fluidic tubes	Biomedical applications	[178]
Pneumatic actuation	CB/PLA composites	Yes	No	With stiffness tunability and intrinsic self-sensing feedback	Cardiac-mimicking actuator and active therapeutic insoles	[194]
Pneumatic actuation	Diels–Alder polymers	Yes	No	To be self-healing	Self-healing and recycled soft robots	[52]
Pneumatic actuation	Ecoflex	Yes	No	With anti-freezing ionic skin to provide deformation/position feedback	Soft robot working at extremely cold conditions	[54]
Pneumatic actuation	Silicone rubber	Yes	No	With a bimodal self-powered flexible sensor and ML- enabled object description	Intelligent soft robotic system that accurately describes objects	[220]
Pneumatic actuation	Dragon Skin 30 solution	Yes	Yes	With two flexible optical waveguide sensors and ML- enabled recognition of 12 contact positions	Soft robots worked in underwater environments	[51]
Pneumatic actuation	Silicone	Yes	No	With sensing of static and dynamic contact, vibration, strain and ML-enabled object recognition and augmented feedback	Device to achieve dual-way and multimodal communication	[235]
